# Supplementary figures and images for: First passage time analysis of spatial mutation patterns reveals sub-clonal evolutionary dynamics in colorectal cancer
Source: PLoS Comput Biol. 2023 Mar 13;19(3):e1010952. doi: 10.1371/journal.pcbi.1010952 (PMC10035892; doi:10.1371/journal.pcbi.1010952)

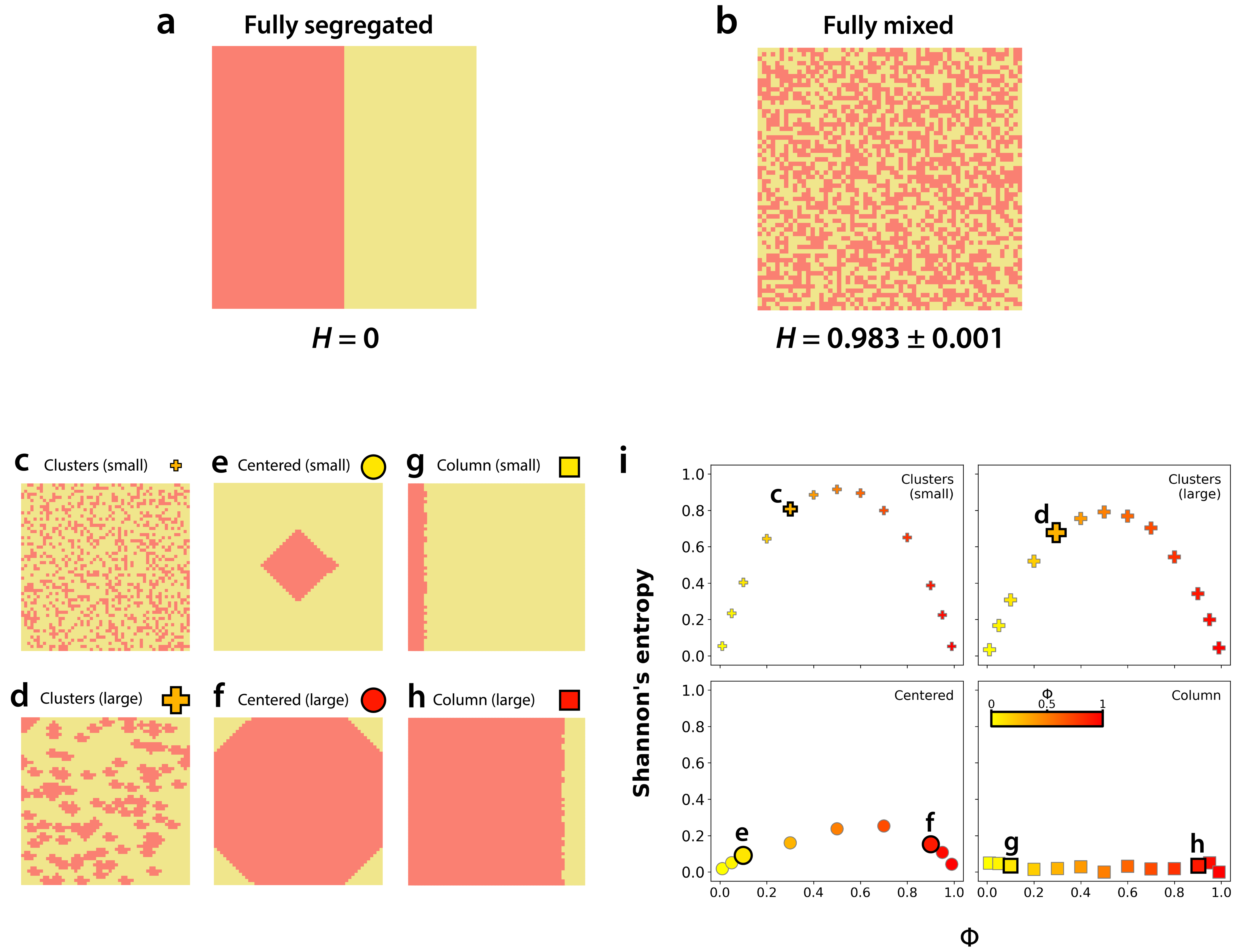

Supplement: S1 Fig — Shannon’s entropy analysis using the approach described in Ref [20]. Validation of our algorithm using (a) fully segregated and (b) fully mixed patterns, where the latter value represents mean ± standard deviation for entropy values of 10 randomly generated fully mixed patterns (ϕ = 0.5 in both cases). (c, d) Shannon’s entropy of patterns generated using the clusters; (e, f) centred and (g, h) column models. (i) Location of the patterns obtained for the three models with varying class ratio, ϕ. All patterns have dimensions of 54 × 54, and Shannon’s entropy was computed using quadrats of size 10 × 10. (TIF) [file pcbi.1010952.s001.tif]

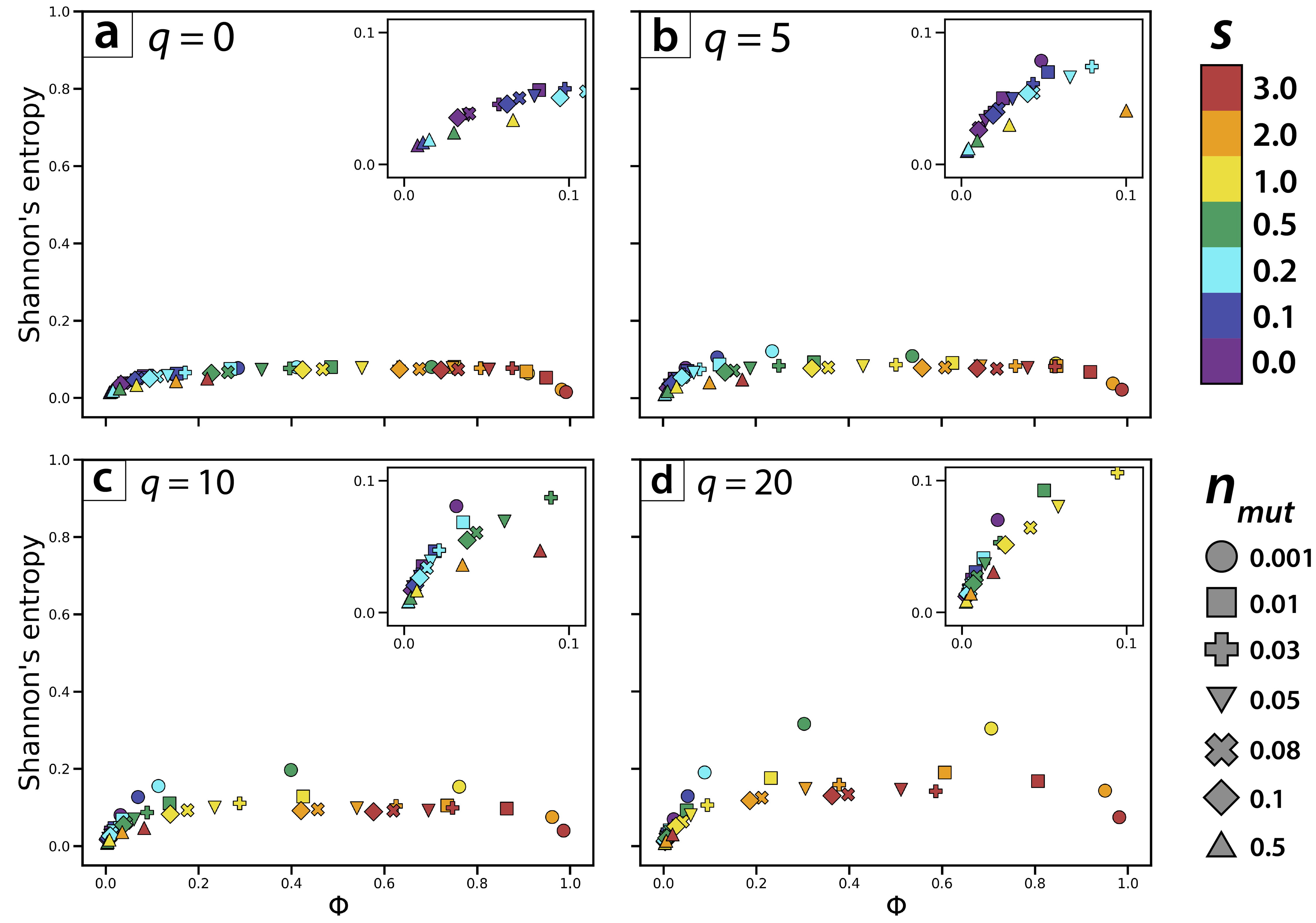

Supplement: S2 Fig — Shannon’s entropy analysis of the same set of simulated tumour sub-clonal patterns as were analysed using CMFPT. Points are coloured according to values of model parameter s and point shape depends on parameter nmut. Data within each panel represent simulations for all possible combinations of s and nmut where s ∈ {0, 0.1, 0.2, 0.5, 1, 2, 3} and nmut ∈ {0.001, 0.01, 0.03, 0.05, 0.08, 0.1, 0.5} (approximately 100 simulated patterns for each parameter combination). Images are separated depending on their pushing value q = 0 (a), q = 5 (b), q = 10 (c) and q = 20 (d). Inset within each panel is a magnified section of the phase space spanning approximately 0 < ϕ < 0.1 and 0 ≤ Shannon’s entropy < 0.1. (TIF) [file pcbi.1010952.s002.tif]

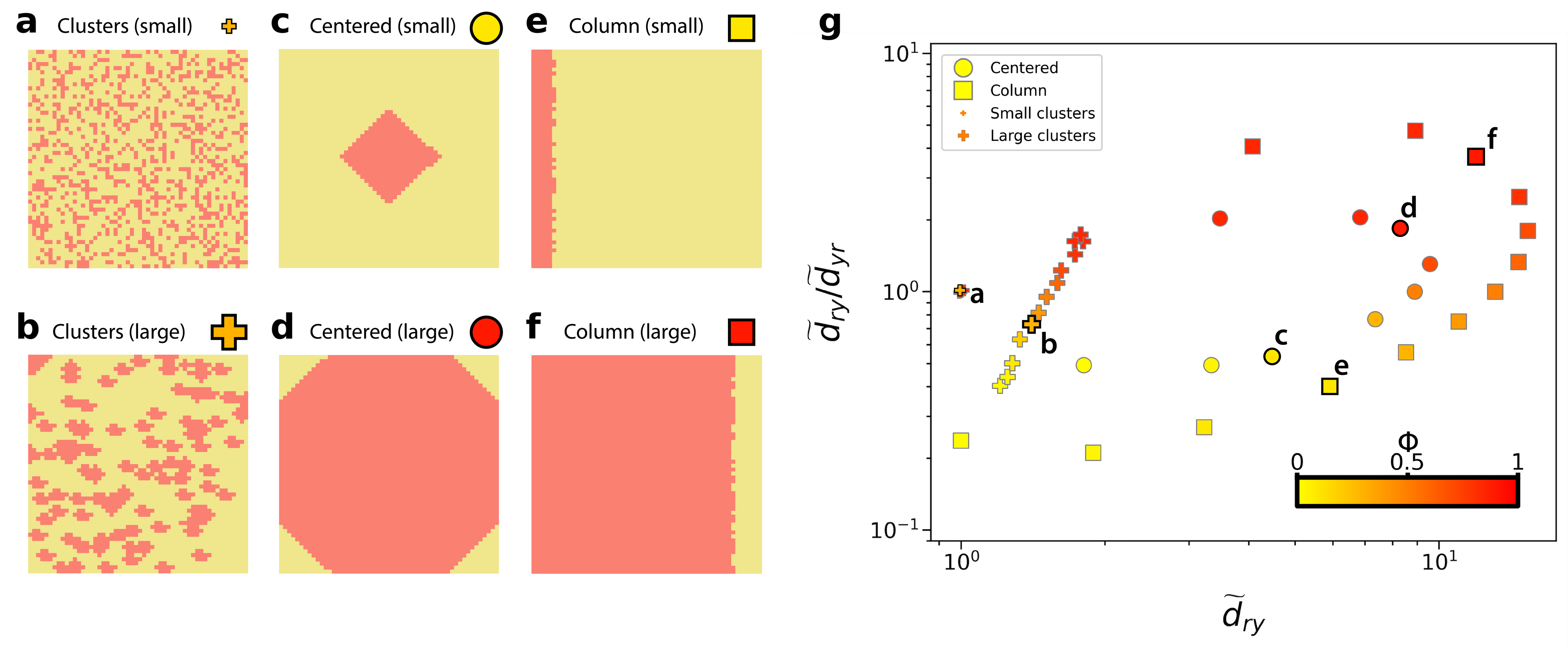

Supplement: S3 Fig — Normalised mean shortest distance analysis of patterns generated using the (a, b) clusters; (c, d) centred and (e, f) column models. (g) Location of the patterns obtained for the three models with varying class ratio, ϕ. Normalised mean shortest distance from red to yellow cells is denoted d˜ry, and from yellow to red denoted d˜yr. (TIF) [file pcbi.1010952.s003.tif]

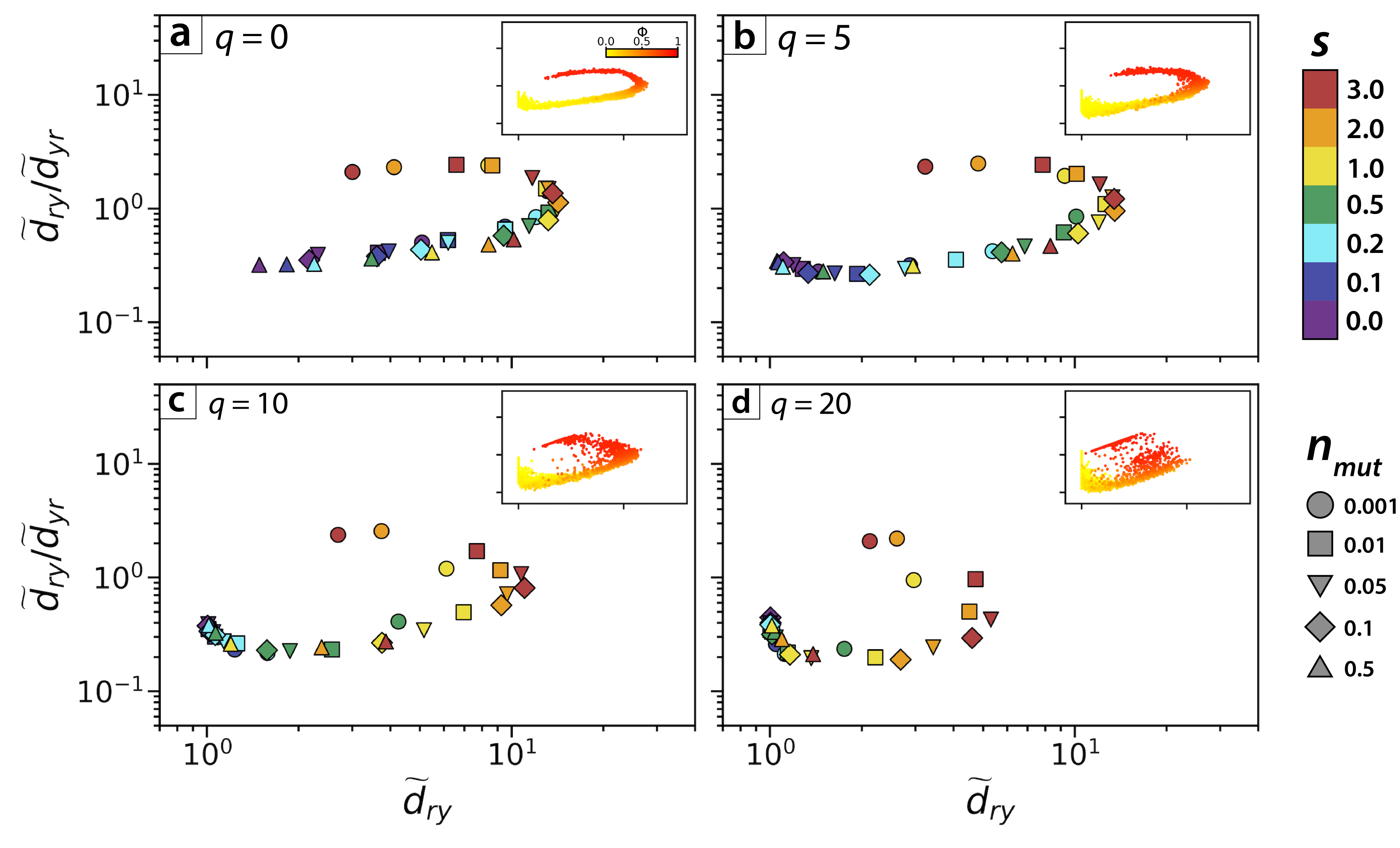

Supplement: S4 Fig — Normalised mean shortest distance analysis of the same set of simulated tumour sub-clonal patterns as were analysed using CMFPT. Points are coloured according to values of model parameter s and point shape depends on parameter nmut. Data within each panel represent simulations for all possible combinations of s and nmut where s ∈ {0, 0.1, 0.2, 0.5, 1, 2, 3} and nmut ∈ {0.001, 0.01, 0.05, 0.1, 0.5} (approximately 100 simulated patterns for each parameter combination). Images are separated depending on their pushing value q = 0 (a), q = 5 (b), q = 10 (c) and q = 20 (d). (TIF) [file pcbi.1010952.s004.tif]

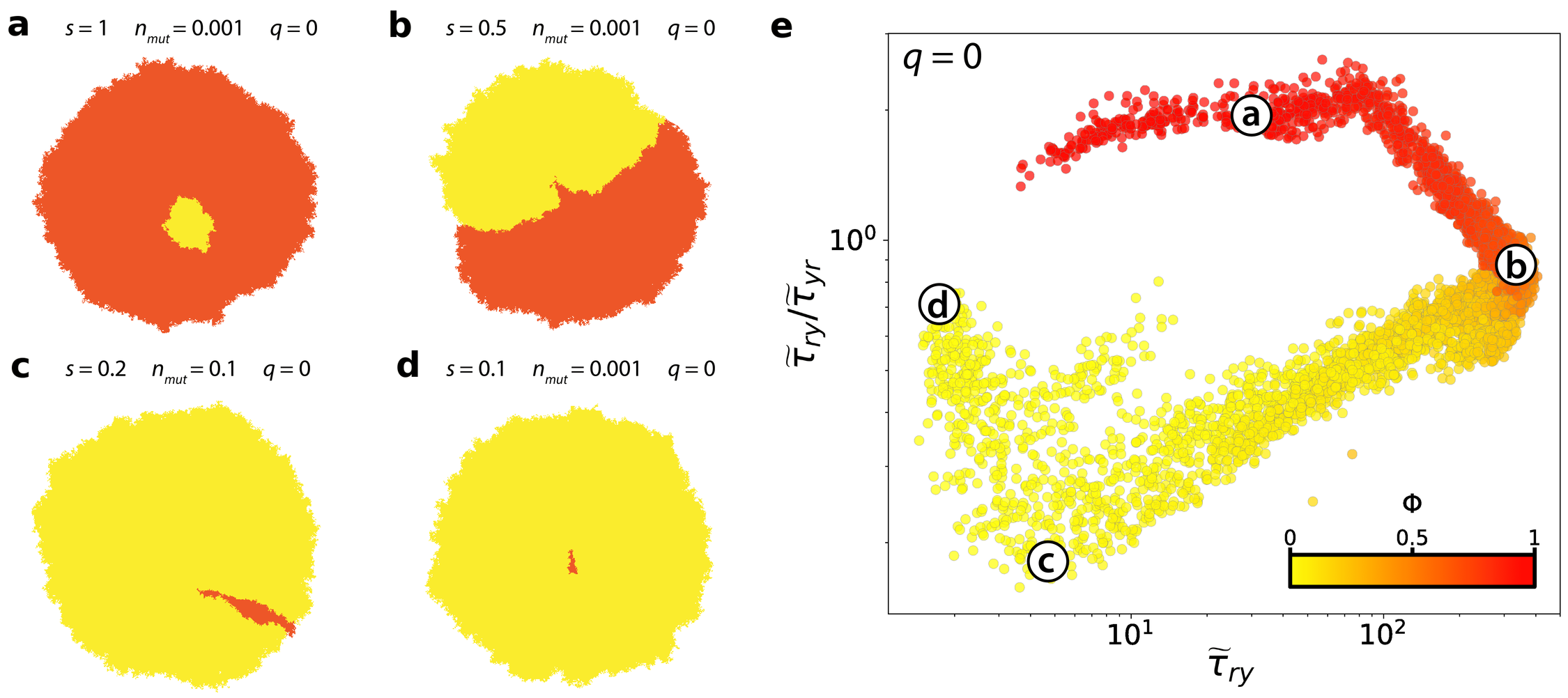

Supplement: S5 Fig — (a-d) Representative examples of tumour sub-clonal patterns simulated with a pushing strength of q = 0. (e) CMFPT analysis of all simulated sub-clonal patterns with a pushing strength of q = 0 in the (τ˜ry,τ˜ry/τ˜yr) phase space. Data represent simulations for all possible combinations of s and nmut where s ∈ {0, 0.1, 0.2, 0.5, 1, 2, 3} and nmut ∈ {0.001, 0.01, 0.03, 0.05, 0.08, 0.1, 0.5}, with q = 0 (approximately 100 simulated patterns for each parameter combination). Points are coloured according to pattern class ratio, ϕ, and images shown in (a-d) are highlighted in the phase space. (TIF) [file pcbi.1010952.s005.tif]

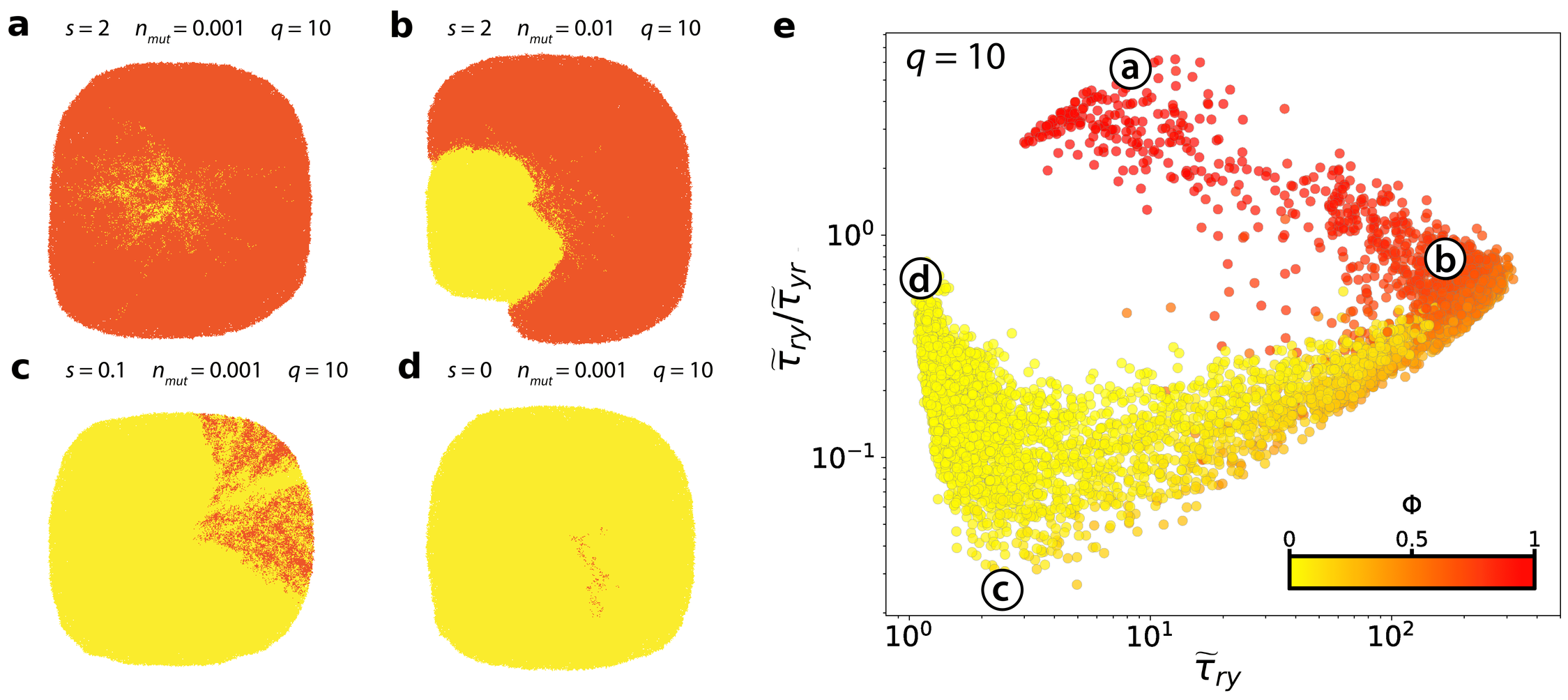

Supplement: S6 Fig — (a-d) Representative examples of tumour sub-clonal patterns simulated with a pushing strength of q = 10. (e) CMFPT analysis of all simulated sub-clonal patterns with a pushing strength of q = 10 in the (τ˜ry,τ˜ry/τ˜yr) phase space. Data represent simulations for all possible combinations of s and nmut where s ∈ {0, 0.1, 0.2, 0.5, 1, 2, 3} and nmut ∈ {0.001, 0.01, 0.03, 0.05, 0.08, 0.1, 0.5}, with q = 10 (approximately 100 simulated patterns for each parameter combination). Points are coloured according to pattern class ratio, ϕ, and images shown in (a-d) are highlighted in the phase space. (TIF) [file pcbi.1010952.s006.tif]

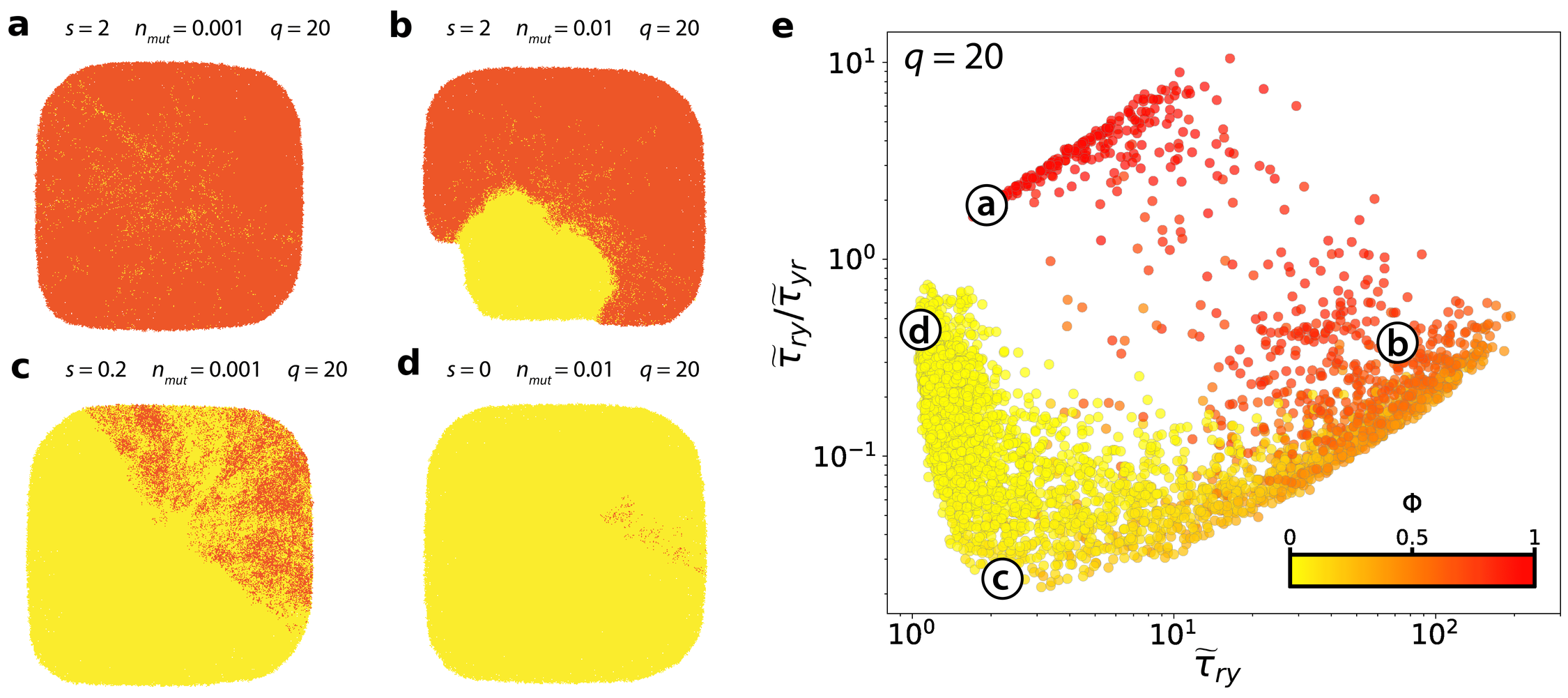

Supplement: S7 Fig — (a-d) Representative examples of tumour sub-clonal patterns simulated with a pushing strength of q = 20. (e) CMFPT analysis of all simulated sub-clonal patterns with a pushing strength of q = 20 in the (τ˜ry,τ˜ry/τ˜yr) phase space. Data represent simulations for all possible combinations of s and nmut where s ∈ {0, 0.1, 0.2, 0.5, 1, 2, 3} and nmut ∈ {0.001, 0.01, 0.03, 0.05, 0.08, 0.1, 0.5}, with q = 20 (approximately 100 simulated patterns for each parameter combination). Points are coloured according to pattern class ratio, ϕ, and images shown in (a-d) are highlighted in the phase space. (TIF) [file pcbi.1010952.s007.tif]

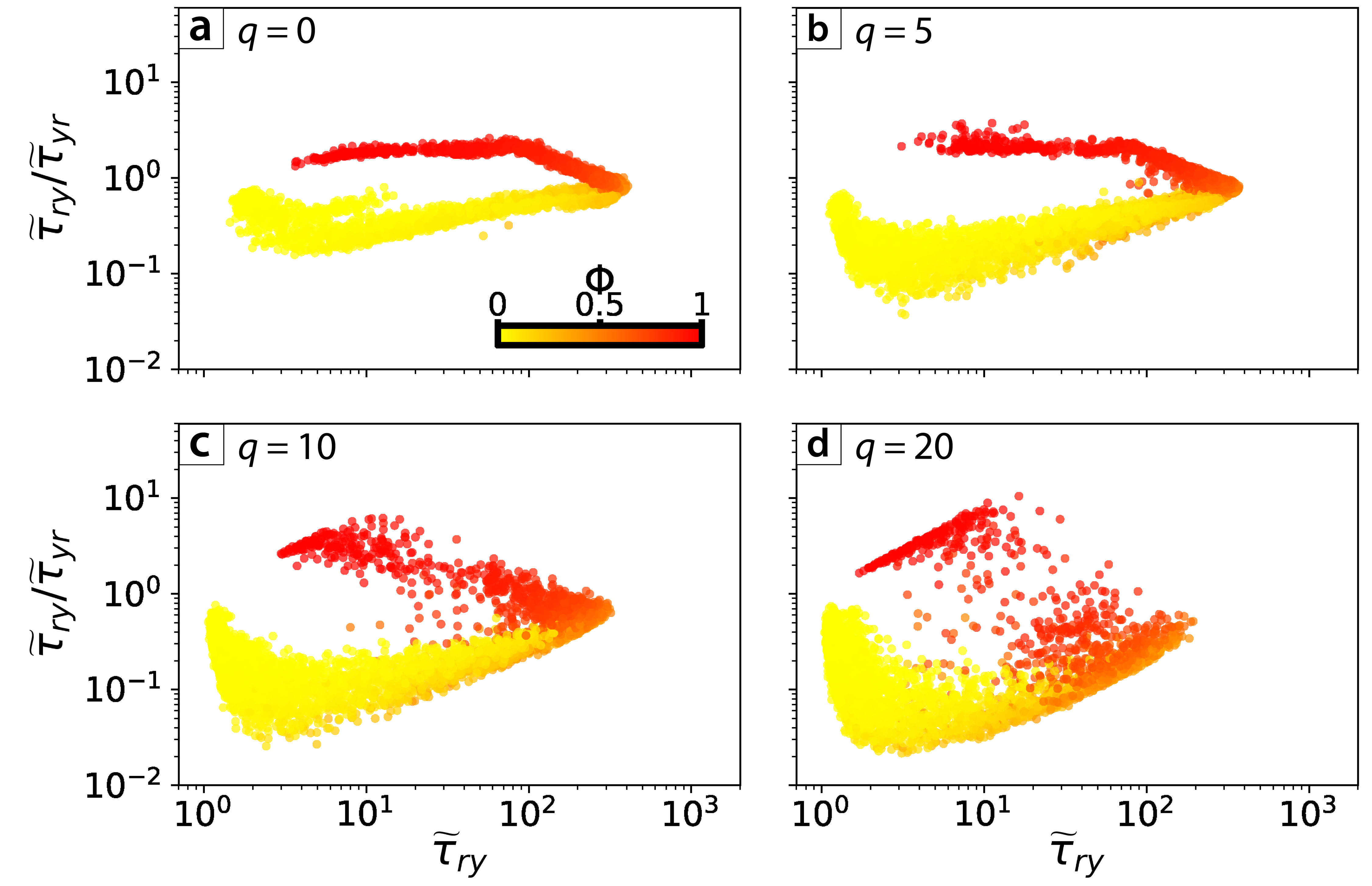

Supplement: S8 Fig — Images generated by the model in the phase space (τ˜ry,τ˜ry/τ˜yr), with points coloured according to value of ϕ. Data within each panel represent simulations for all possible combinations of s and nmut where s ∈ {0, 0.1, 0.2, 0.5, 1, 2, 3} and nmut ∈ {0.001, 0.01, 0.03, 0.05, 0.08, 0.1, 0.5} (approximately 100 simulated patterns for each parameter combination). Images are separated depending on their pushing value (a) 0, (b) 5, (c) 10 and (d) 20. (TIF) [file pcbi.1010952.s008.tif]

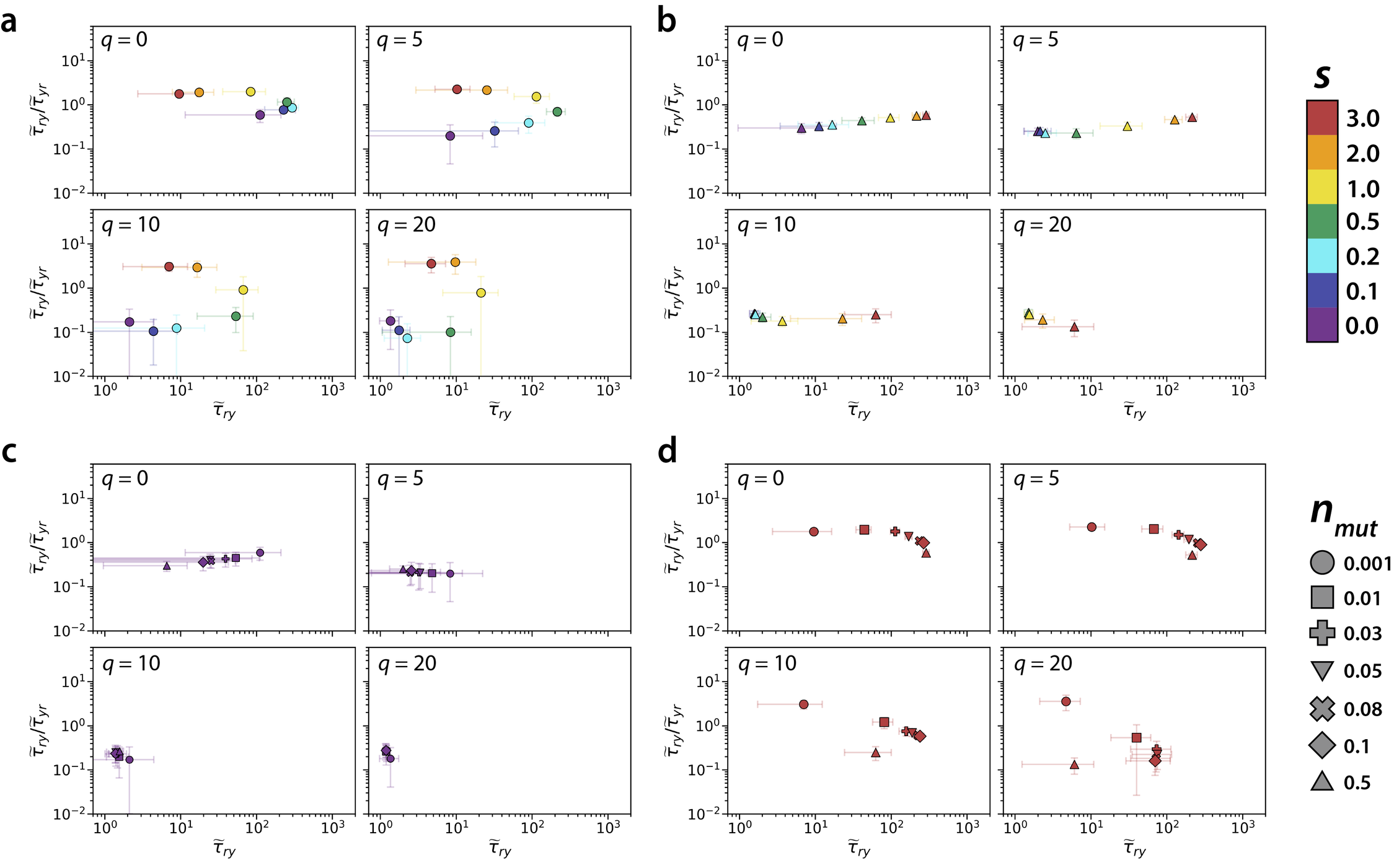

Supplement: S9 Fig — Representative data showing distribution (mean and variance) of CMFPT measured in simulated sub-clonal patterns for various s, nmut and q combinations. For each combination of parameters, we simulated approximately 100 tumours and quantified the sub-clonal patterns using CMFPT. Top row: data for (a) nmut = 0.001 and (b) nmut = 0.5 tumour, both with s ∈ {0, 0.1, 0.2, 0.5, 1, 2, 3}. Bottom row: data for (c) s = 0 and (d) s = 3 tumours, both with nmut ∈ {0.001, 0.01, 0.03, 0.05, 0.08, 0.1, 0.5}. Data are mean ± s.d. (TIF) [file pcbi.1010952.s009.tif]

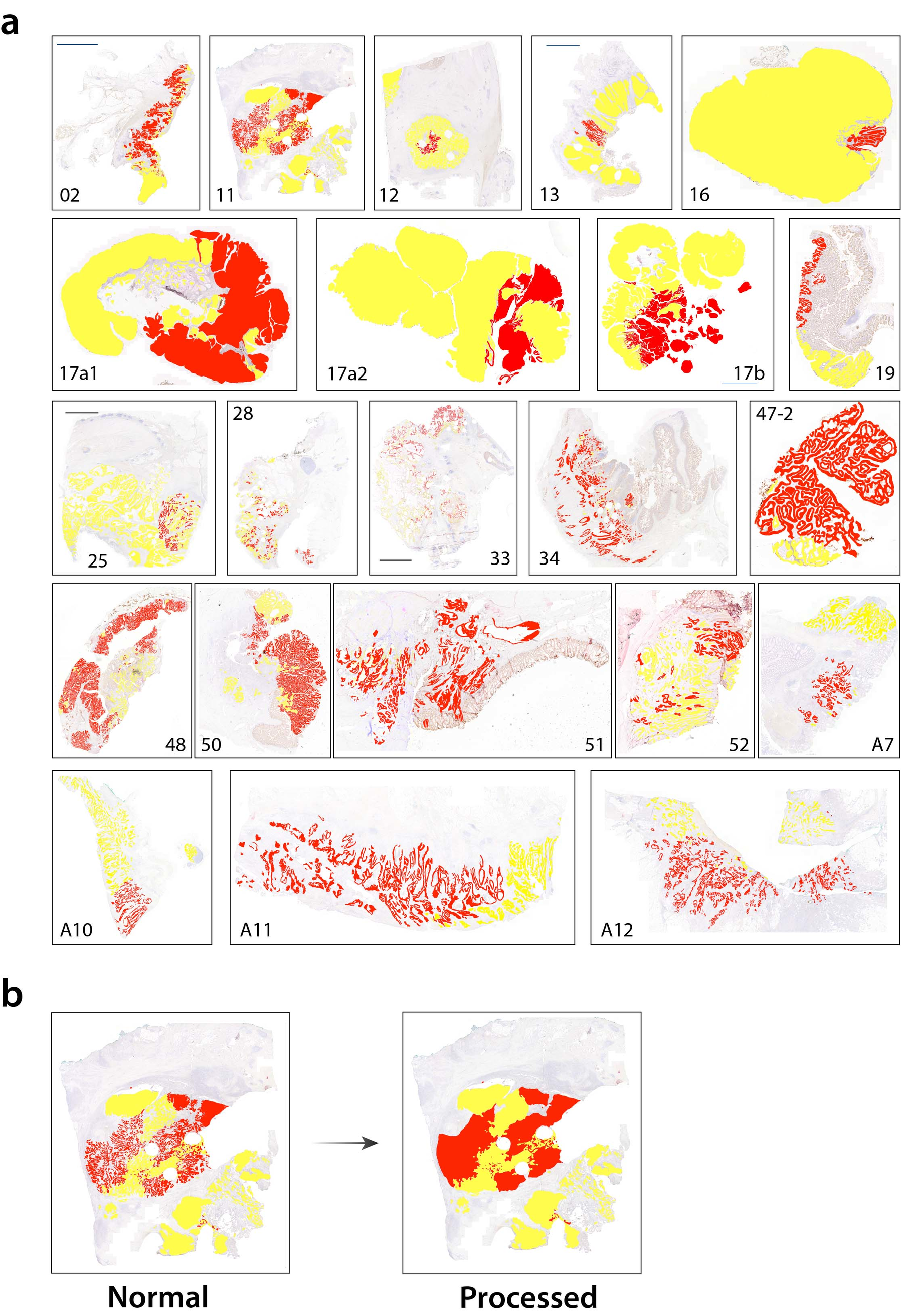

Supplement: S10 Fig — (a) Sub-clonal mutations in colorectal tumours analysed with BaseScope used in this study, from Refs [20, 33]. Colorectal tumour cells and surrounding epithelium is pictured, with wild-type tumour cells highlighted in yellow, and mutated sub-clonal tumour population highlighted in red. Non-cancerous tissue is not highlighted. Scale bars, where present, represent 2000μm (b) Representative example of a “raw” BaseScope image, and the same image after application of the pre-processing steps described in the main text. (TIF) [file pcbi.1010952.s010.tif]

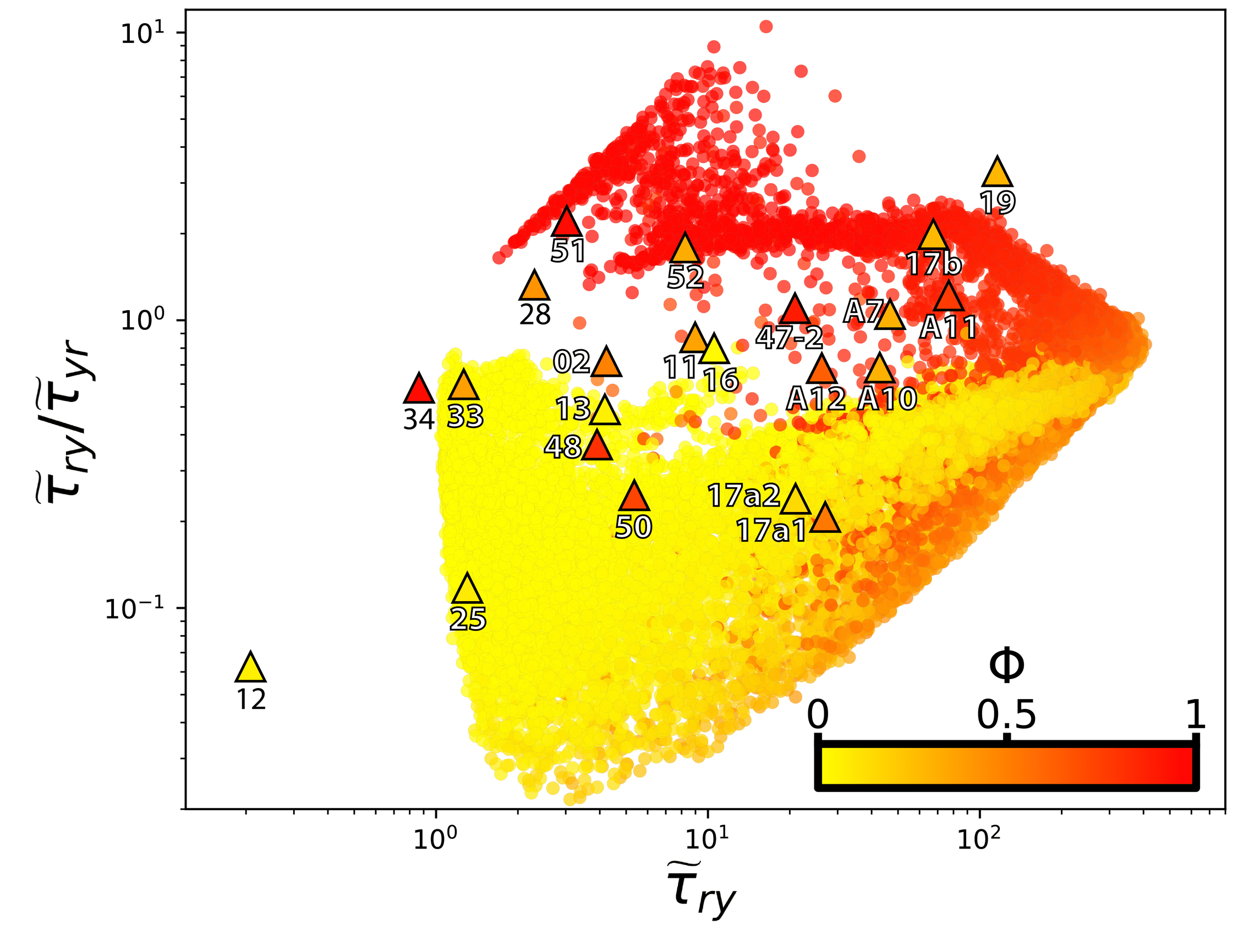

Supplement: S11 Fig — CMFPT analysis of human colorectal tumour samples plotted in the (τ˜ry,τ˜ry/τ˜yr) phase space. Each triangular marker represents a single colorectal cancer sample analysed with BaseScope. Circular points represent all simulated sub-clonal patterns, and points are coloured according to pattern class ratio, ϕ. (TIF) [file pcbi.1010952.s011.tif]

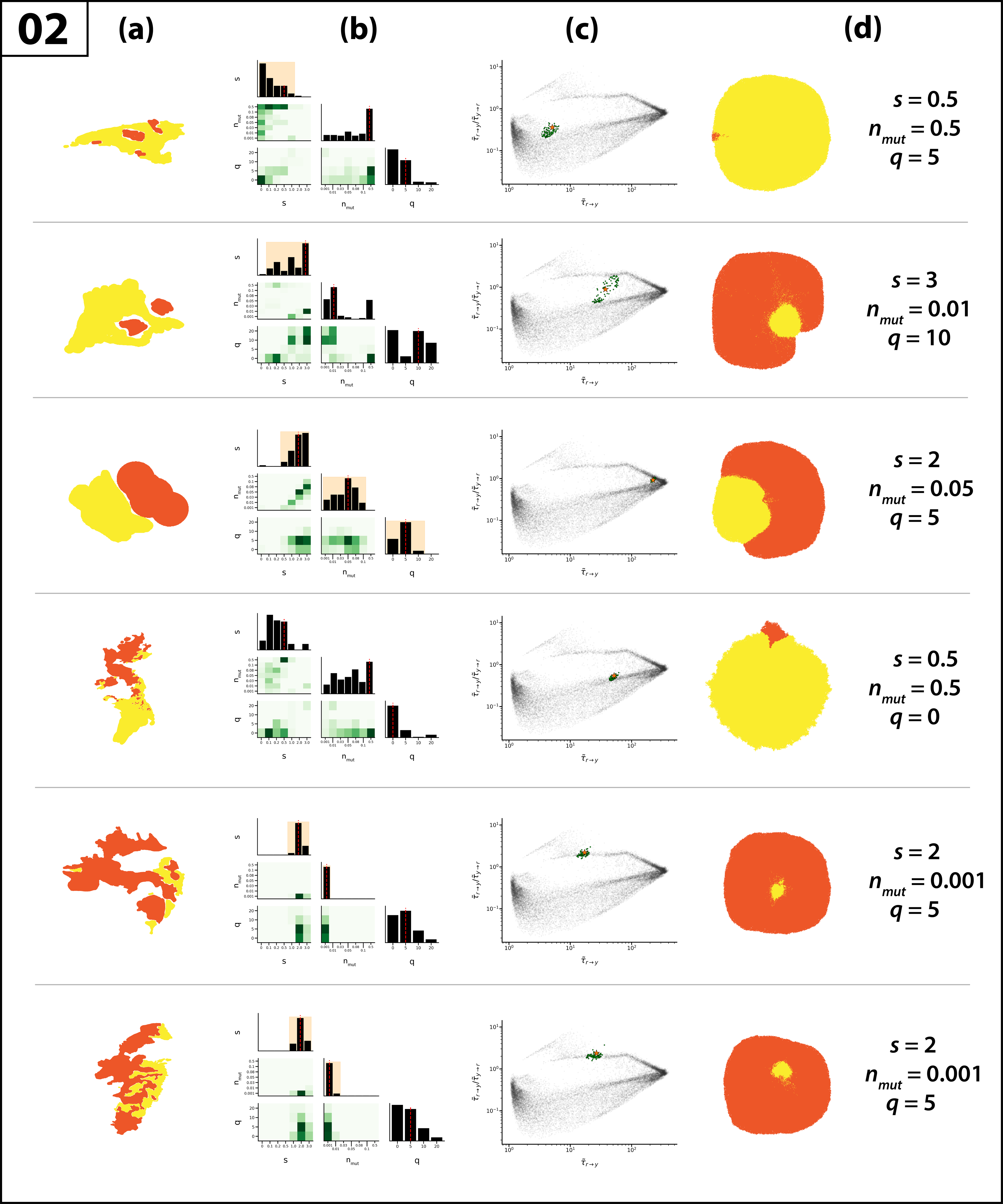

Supplement: S12 Fig — (a) Sub-sample of sample 02. (b) Marginal posterior distributions of model parameters s, nmut, and q, representing mutant selection strength, mutation timing and cell pushing strength respectively. Inferred parameter value is indicated by the vertical dashed line along the diagonal panels. 95% credible regions lie within the shaded region in the diagonal panels. Where no shaded region is given, this interval was the entire parameter range. (c) All analysed simulated sub-clonal mixing patterns (grey points) with CMFPT value of the BaseScope sub-sample (star) and posterior samples (green points). (d) Best-fit simulated sub-clonal pattern and parameters representing the most abundant parameter combination within the posterior distribution. (TIF) [file pcbi.1010952.s012.tif]

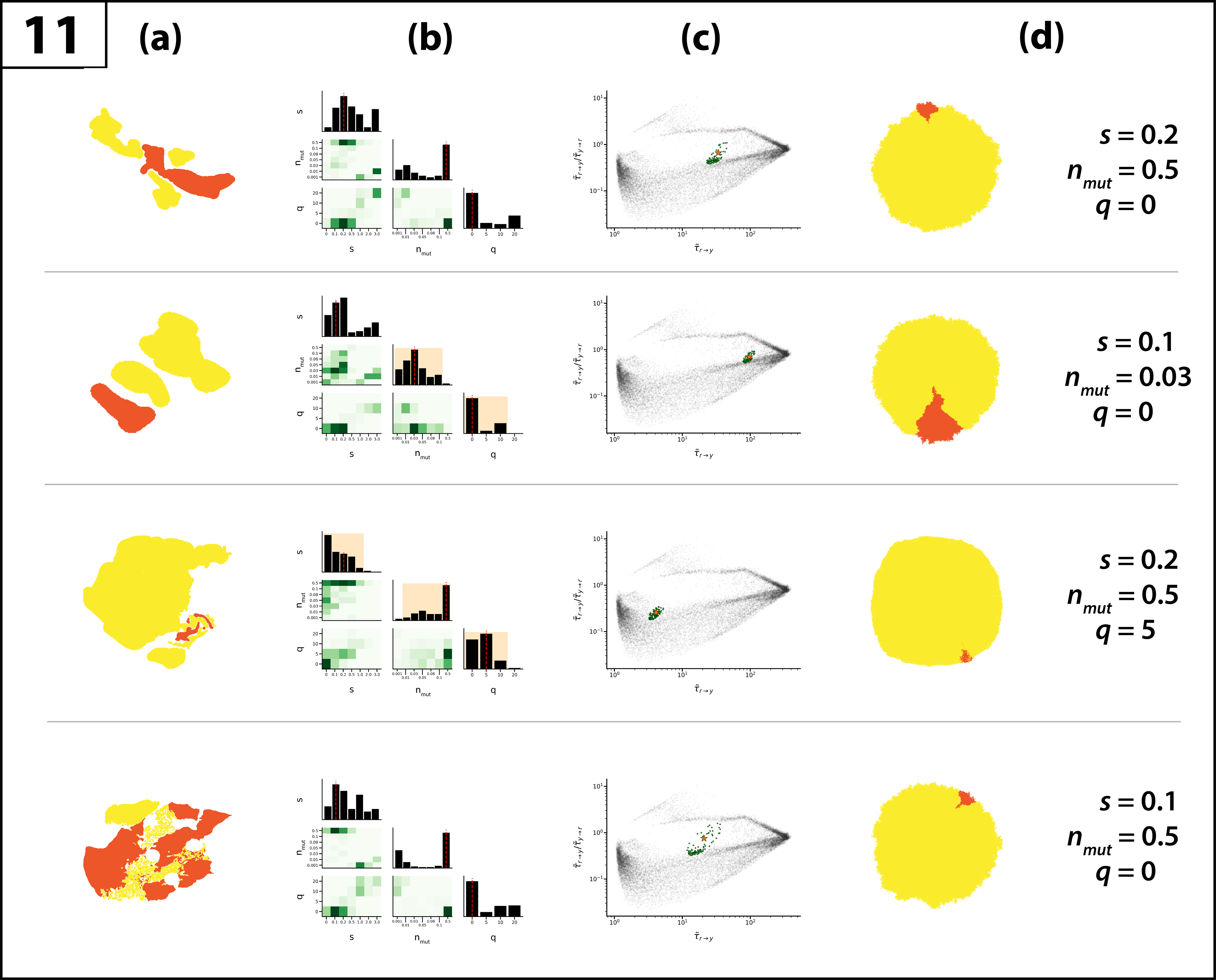

Supplement: S13 Fig — (a) Sub-sample of sample 11. (b) Marginal posterior distributions of model parameters s, nmut, and q, representing mutant selection strength, mutation timing and cell pushing strength respectively. Inferred parameter value is indicated by the vertical dashed line along the diagonal panels. 95% credible regions lie within the shaded region in the diagonal panels. Where no shaded region is given, this interval was the entire parameter range. (c) All analysed simulated sub-clonal mixing patterns (grey points) with CMFPT value of the BaseScope sub-sample (star) and posterior samples (green points). (d) Best-fit simulated sub-clonal pattern and parameters representing the most abundant parameter combination within the posterior distribution. (TIF) [file pcbi.1010952.s013.tif]

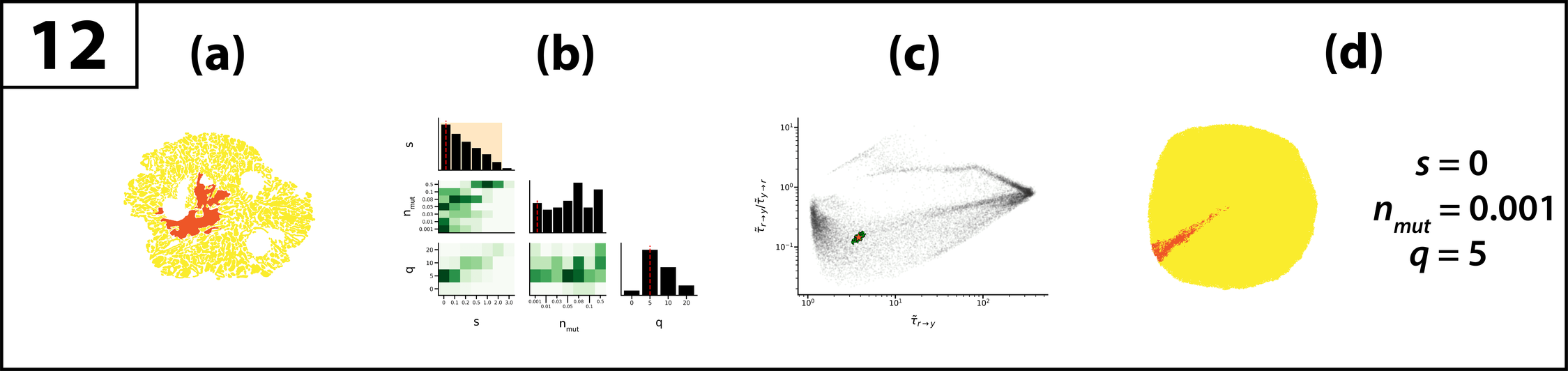

Supplement: S14 Fig — (a) Sub-sample of sample 12. (b) Marginal posterior distributions of model parameters s, nmut, and q, representing mutant selection strength, mutation timing and cell pushing strength respectively. Inferred parameter value is indicated by the vertical dashed line along the diagonal panels. 95% credible regions lie within the shaded region in the diagonal panels. Where no shaded region is given, this interval was the entire parameter range. (c) All analysed simulated sub-clonal mixing patterns (grey points) with CMFPT value of the BaseScope sub-sample (star) and posterior samples (green points). (d) Best-fit simulated sub-clonal pattern and parameters representing the most abundant parameter combination within the posterior distribution. (TIF) [file pcbi.1010952.s014.tif]

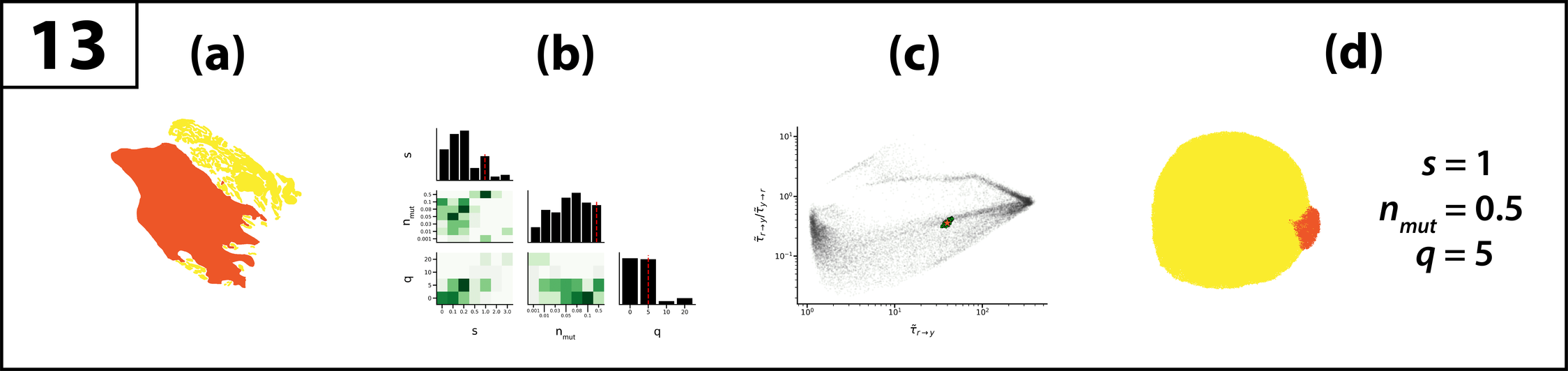

Supplement: S15 Fig — (a) Sub-sample of sample 13. (b) Marginal posterior distributions of model parameters s, nmut, and q, representing mutant selection strength, mutation timing and cell pushing strength respectively. Inferred parameter value is indicated by the vertical dashed line along the diagonal panels. 95% credible regions lie within the shaded region in the diagonal panels. Where no shaded region is given, this interval was the entire parameter range. (c) All analysed simulated sub-clonal mixing patterns (grey points) with CMFPT value of the BaseScope sub-sample (star) and posterior samples (green points). (d) Best-fit simulated sub-clonal pattern and parameters representing the most abundant parameter combination within the posterior distribution. (TIF) [file pcbi.1010952.s015.tif]

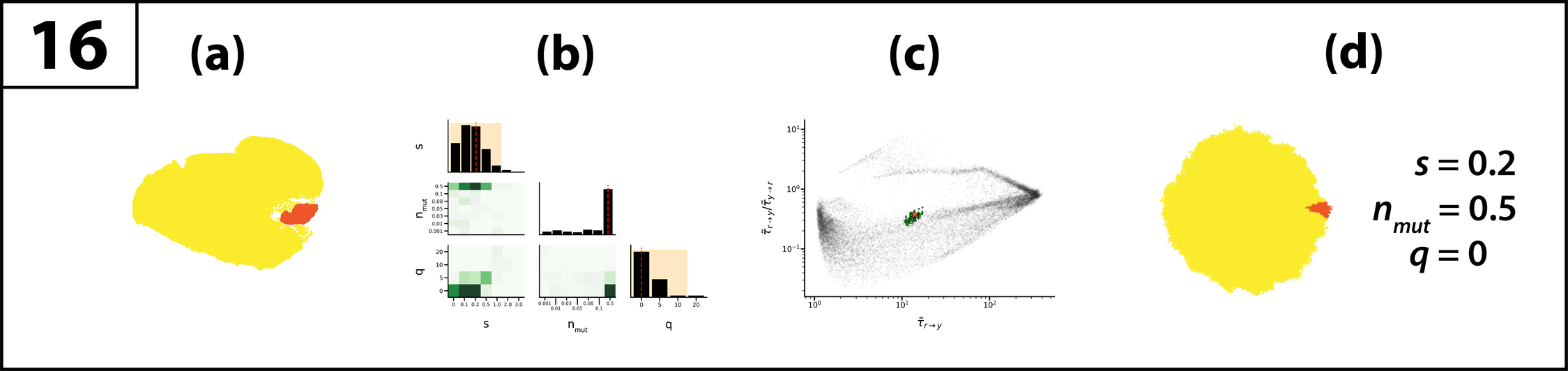

Supplement: S16 Fig — (a) Sub-sample of sample 16. (b) Marginal posterior distributions of model parameters s, nmut, and q, representing mutant selection strength, mutation timing and cell pushing strength respectively. Inferred parameter value is indicated by the vertical dashed line along the diagonal panels. 95% credible regions lie within the shaded region in the diagonal panels. Where no shaded region is given, this interval was the entire parameter range. (c) All analysed simulated sub-clonal mixing patterns (grey points) with CMFPT value of the BaseScope sub-sample (star) and posterior samples (green points). (d) Best-fit simulated sub-clonal pattern and parameters representing the most abundant parameter combination within the posterior distribution. (TIF) [file pcbi.1010952.s016.tif]

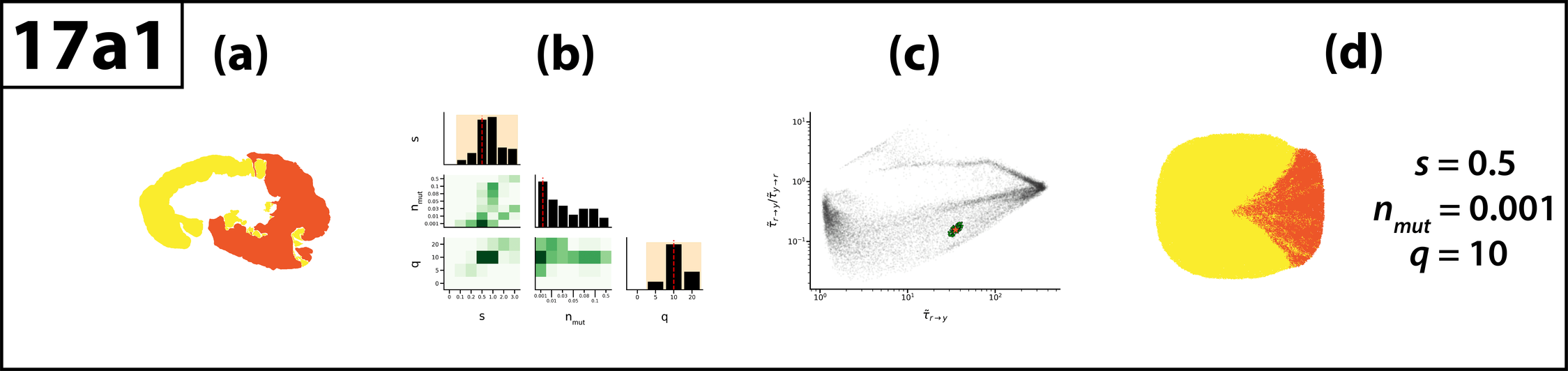

Supplement: S17 Fig — (a) Sub-sample of sample 17a1. (b) Marginal posterior distributions of model parameters s, nmut, and q, representing mutant selection strength, mutation timing and cell pushing strength respectively. Inferred parameter value is indicated by the vertical dashed line along the diagonal panels. 95% credible regions lie within the shaded region in the diagonal panels. Where no shaded region is given, this interval was the entire parameter range. (c) All analysed simulated sub-clonal mixing patterns (grey points) with CMFPT value of the BaseScope sub-sample (star) and posterior samples (green points). (d) Best-fit simulated sub-clonal pattern and parameters representing the most abundant parameter combination within the posterior distribution. (TIF) [file pcbi.1010952.s017.tif]

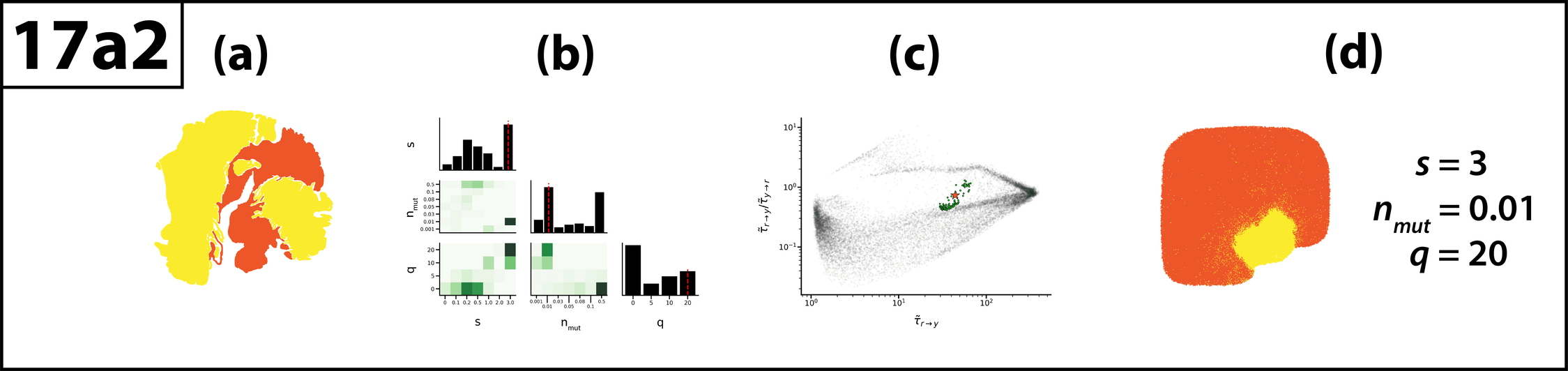

Supplement: S18 Fig — (a) Sub-sample of sample 17a2. (b) Marginal posterior distributions of model parameters s, nmut, and q, representing mutant selection strength, mutation timing and cell pushing strength respectively. Inferred parameter value is indicated by the vertical dashed line along the diagonal panels. 95% credible regions lie within the shaded region in the diagonal panels. Where no shaded region is given, this interval was the entire parameter range. (c) All analysed simulated sub-clonal mixing patterns (grey points) with CMFPT value of the BaseScope sub-sample (star) and posterior samples (green points). (d) Best-fit simulated sub-clonal pattern and parameters representing the most abundant parameter combination within the posterior distribution. (TIF) [file pcbi.1010952.s018.tif]

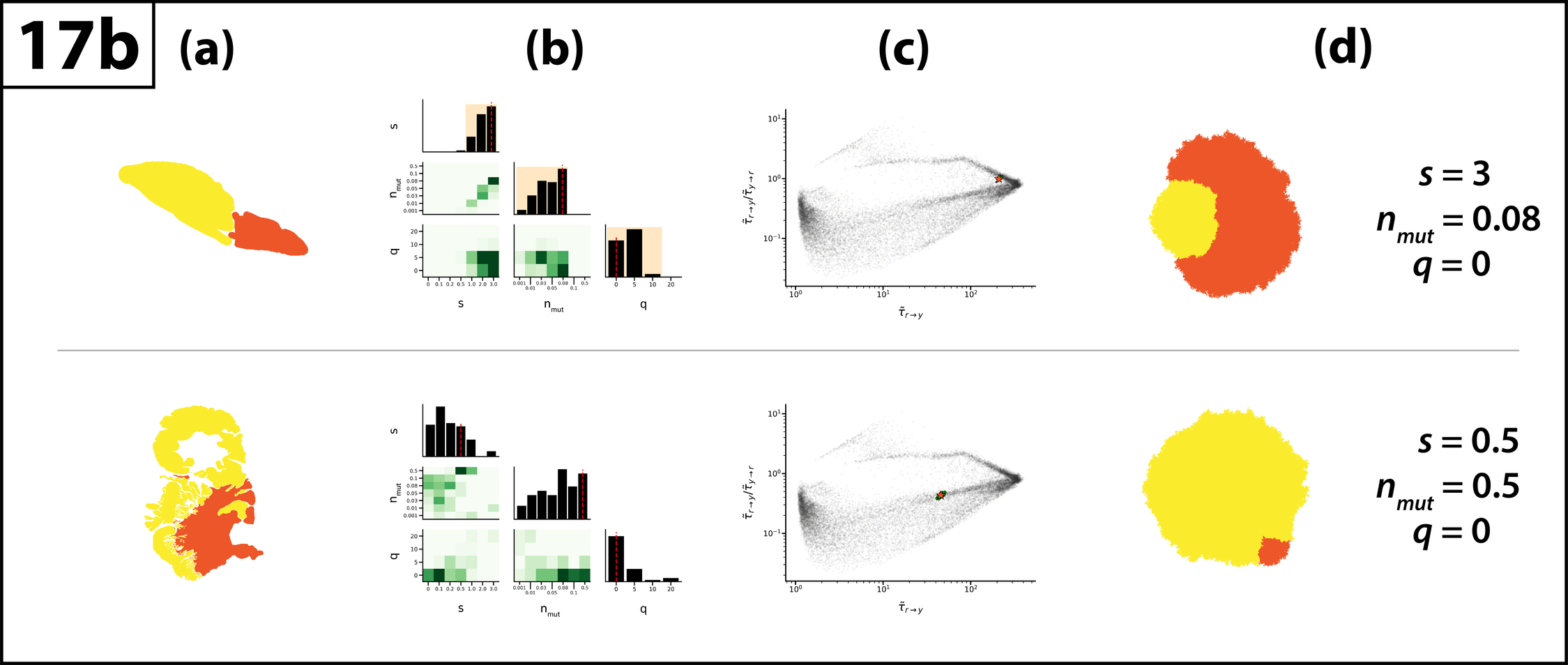

Supplement: S19 Fig — (a) Sub-sample of sample 17b. (b) Marginal posterior distributions of model parameters s, nmut, and q, representing mutant selection strength, mutation timing and cell pushing strength respectively. Inferred parameter value is indicated by the vertical dashed line along the diagonal panels. 95% credible regions lie within the shaded region in the diagonal panels. Where no shaded region is given, this interval was the entire parameter range. (c) All analysed simulated sub-clonal mixing patterns (grey points) with CMFPT value of the BaseScope sub-sample (star) and posterior samples (green points). (d) Best-fit simulated sub-clonal pattern and parameters representing the most abundant parameter combination within the posterior distribution. (TIF) [file pcbi.1010952.s019.tif]

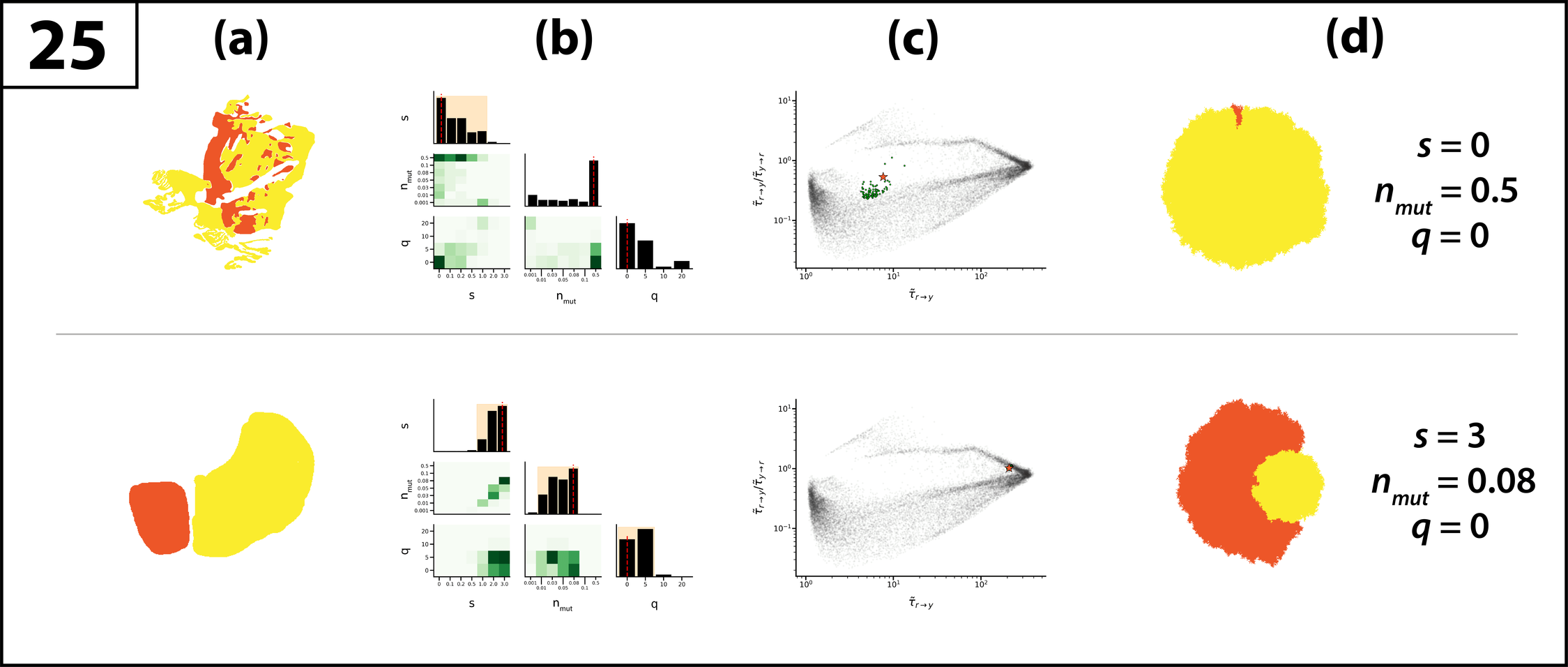

Supplement: S20 Fig — (a) Sub-sample of sample 25. (b) Marginal posterior distributions of model parameters s, nmut, and q, representing mutant selection strength, mutation timing and cell pushing strength respectively. Inferred parameter value is indicated by the vertical dashed line along the diagonal panels. 95% credible regions lie within the shaded region in the diagonal panels. Where no shaded region is given, this interval was the entire parameter range. (c) All analysed simulated sub-clonal mixing patterns (grey points) with CMFPT value of the BaseScope sub-sample (star) and posterior samples (green points). (d) Best-fit simulated sub-clonal pattern and parameters representing the most abundant parameter combination within the posterior distribution. (TIF) [file pcbi.1010952.s020.tif]

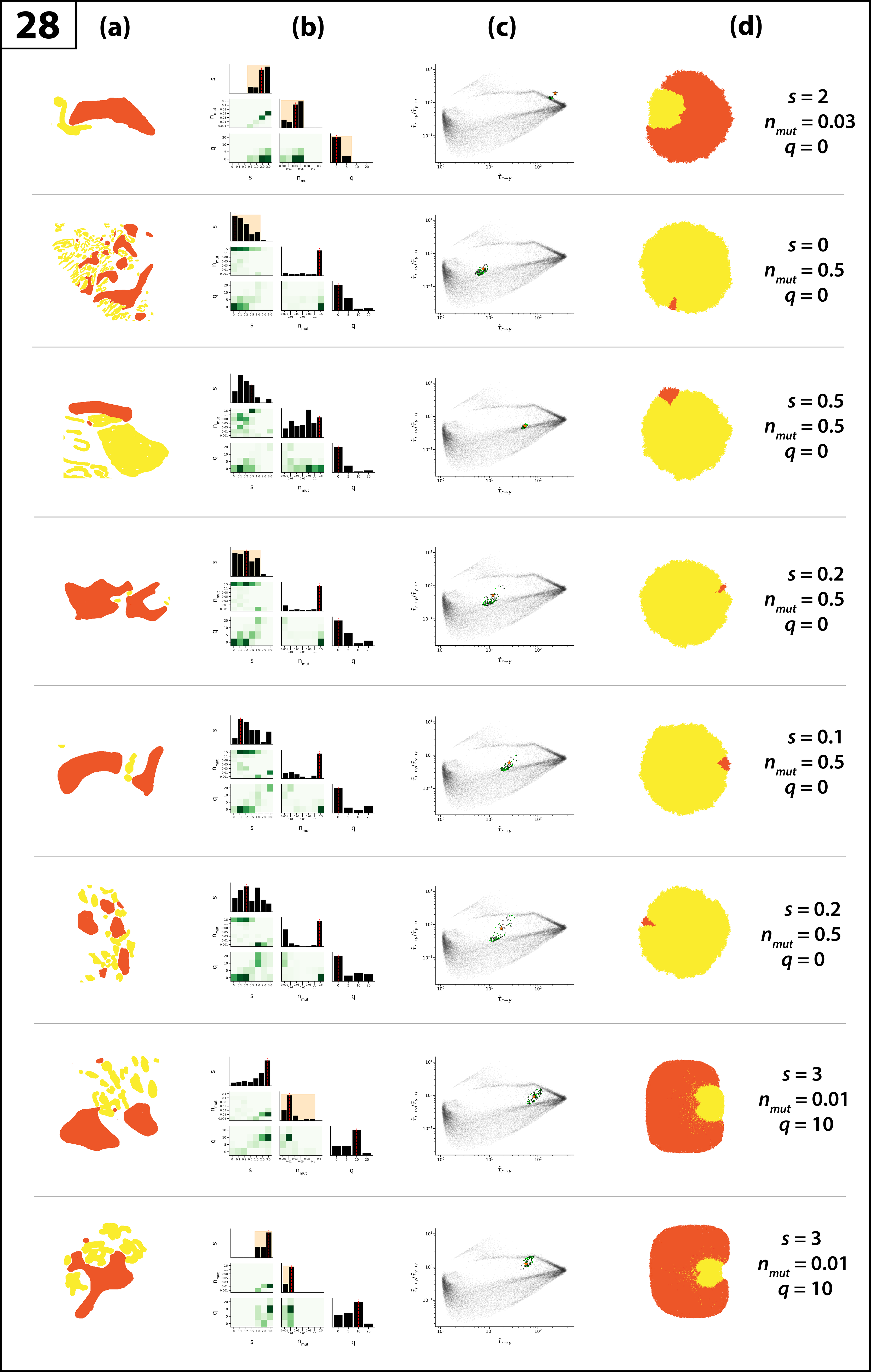

Supplement: S21 Fig — (a) Sub-sample of sample 28. (b) Marginal posterior distributions of model parameters s, nmut, and q, representing mutant selection strength, mutation timing and cell pushing strength respectively. Inferred parameter value is indicated by the vertical dashed line along the diagonal panels. 95% credible regions lie within the shaded region in the diagonal panels. Where no shaded region is given, this interval was the entire parameter range. (c) All analysed simulated sub-clonal mixing patterns (grey points) with CMFPT value of the BaseScope sub-sample (star) and posterior samples (green points). (d) Best-fit simulated sub-clonal pattern and parameters representing the most abundant parameter combination within the posterior distribution. (TIF) [file pcbi.1010952.s021.tif]

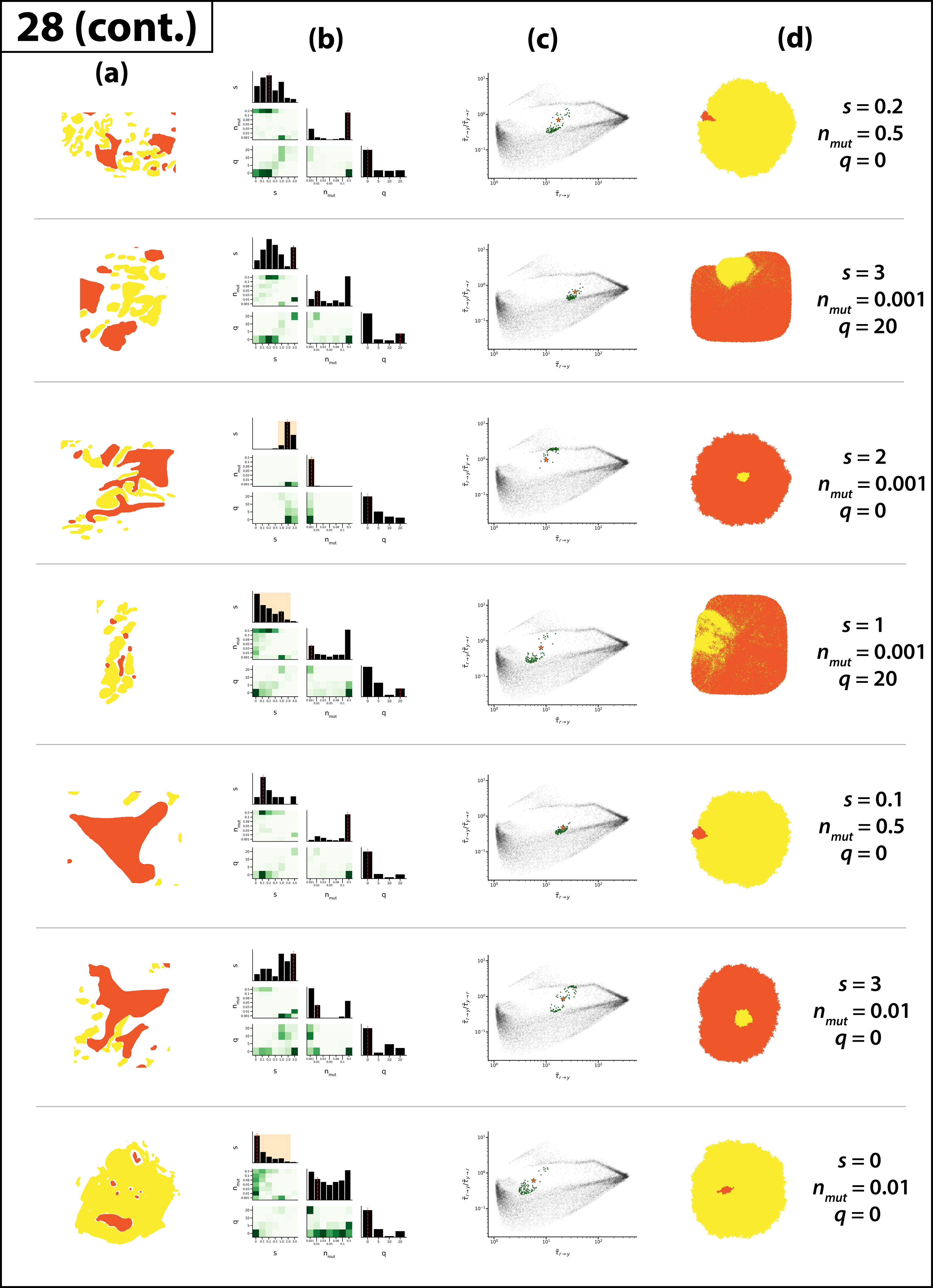

Supplement: S22 Fig — (a) Sub-sample of sample 28. (b) Marginal posterior distributions of model parameters s, nmut, and q, representing mutant selection strength, mutation timing and cell pushing strength respectively. Inferred parameter value is indicated by the vertical dashed line along the diagonal panels. 95% credible regions lie within the shaded region in the diagonal panels. Where no shaded region is given, this interval was the entire parameter range. (c) All analysed simulated sub-clonal mixing patterns (grey points) with CMFPT value of the BaseScope sub-sample (star) and posterior samples (green points). (d) Best-fit simulated sub-clonal pattern and parameters representing the most abundant parameter combination within the posterior distribution. (TIF) [file pcbi.1010952.s022.tif]

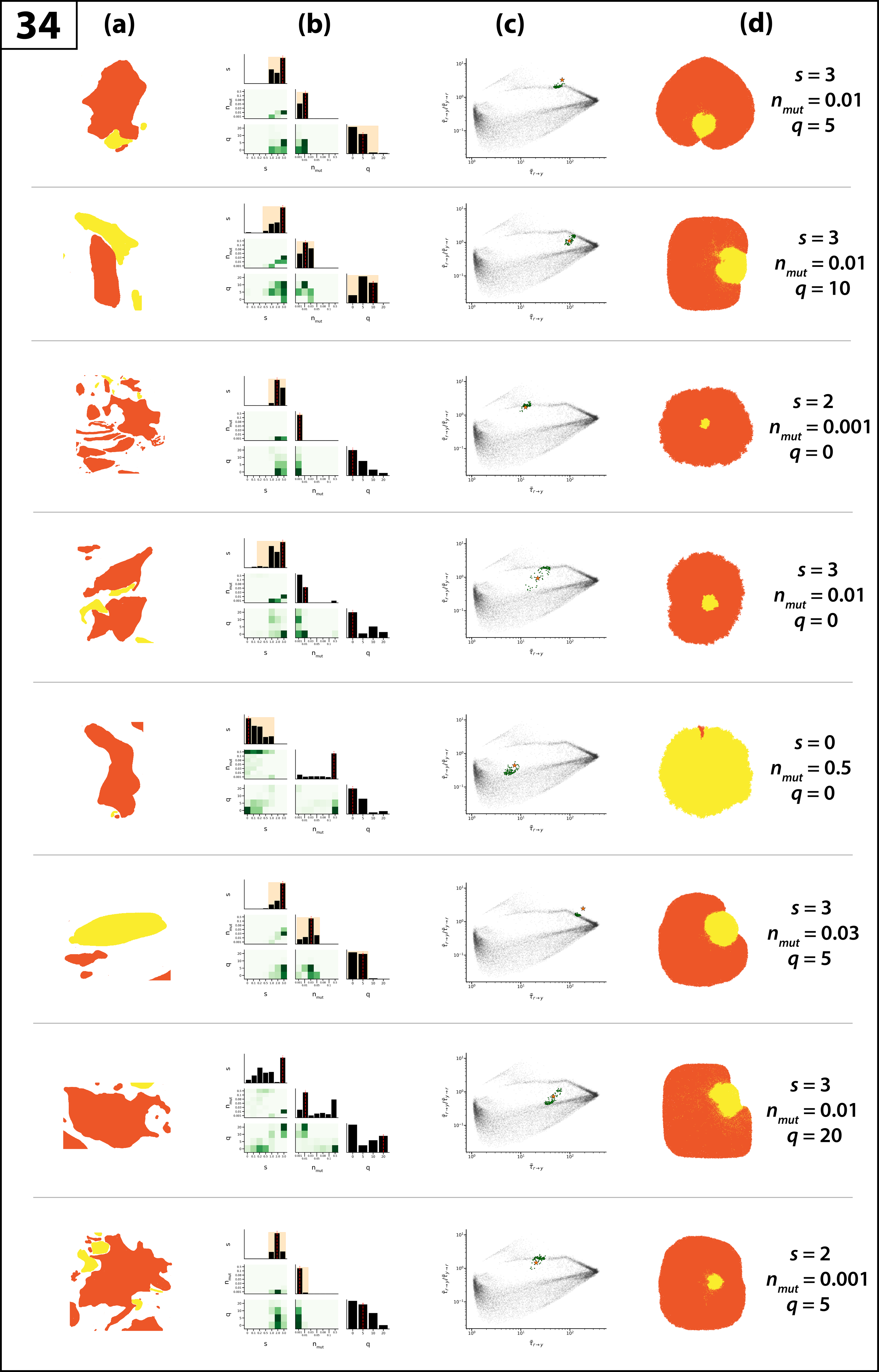

Supplement: S23 Fig — (a) Sub-sample of sample 34. (b) Marginal posterior distributions of model parameters s, nmut, and q, representing mutant selection strength, mutation timing and cell pushing strength respectively. Inferred parameter value is indicated by the vertical dashed line along the diagonal panels. 95% credible regions lie within the shaded region in the diagonal panels. Where no shaded region is given, this interval was the entire parameter range. (c) All analysed simulated sub-clonal mixing patterns (grey points) with CMFPT value of the BaseScope sub-sample (star) and posterior samples (green points). (d) Best-fit simulated sub-clonal pattern and parameters representing the most abundant parameter combination within the posterior distribution. (TIF) [file pcbi.1010952.s023.tif]

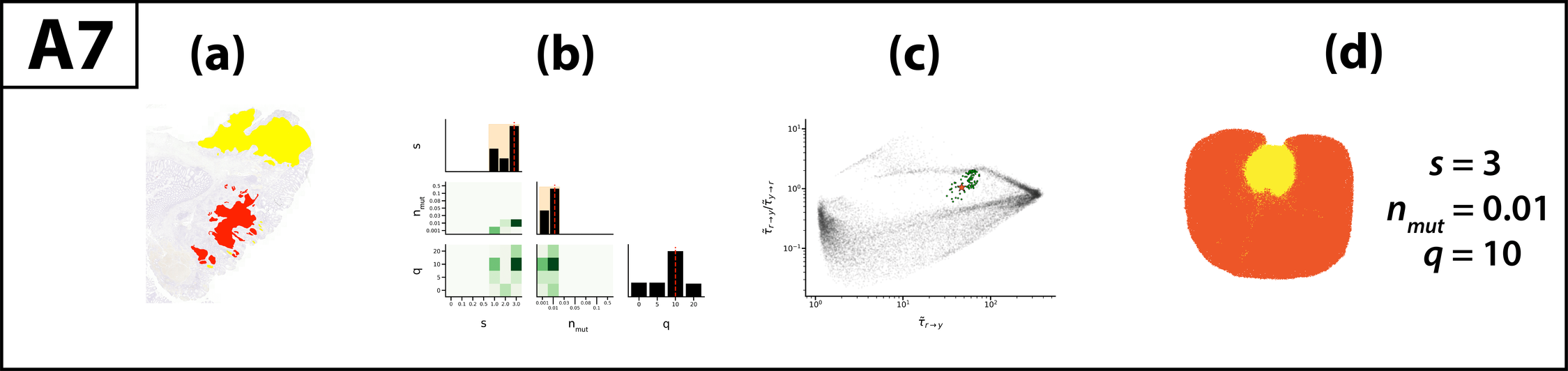

Supplement: S24 Fig — (a) Sub-sample of sample A7. (b) Marginal posterior distributions of model parameters s, nmut, and q, representing mutant selection strength, mutation timing and cell pushing strength respectively. Inferred parameter value is indicated by the vertical dashed line along the diagonal panels. 95% credible regions lie within the shaded region in the diagonal panels. Where no shaded region is given, this interval was the entire parameter range. (c) All analysed simulated sub-clonal mixing patterns (grey points) with CMFPT value of the BaseScope sub-sample (star) and posterior samples (green points). (d) Best-fit simulated sub-clonal pattern and parameters representing the most abundant parameter combination within the posterior distribution. (TIF) [file pcbi.1010952.s024.tif]

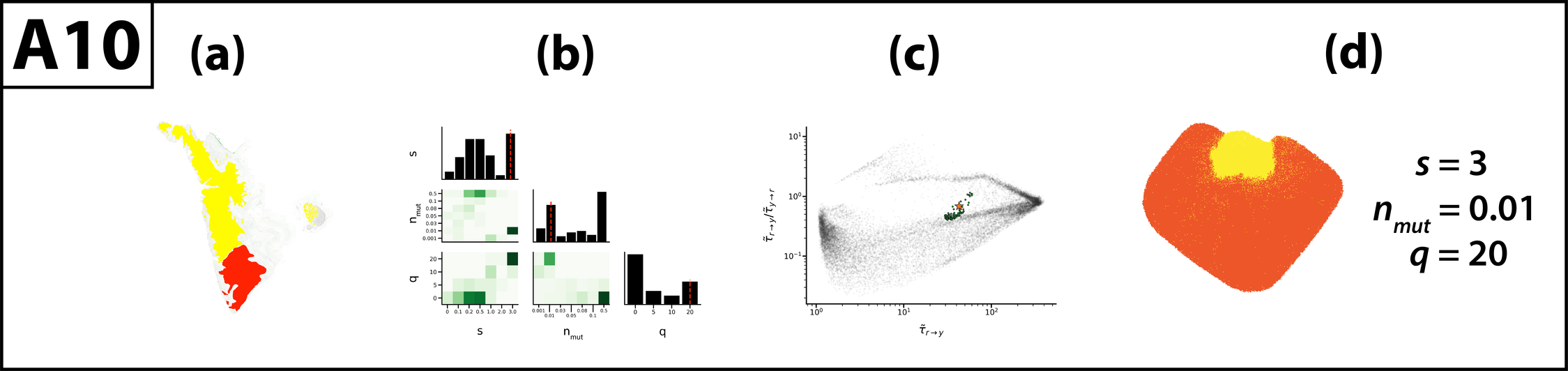

Supplement: S25 Fig — (a) Sub-sample of sample A10. (b) Marginal posterior distributions of model parameters s, nmut, and q, representing mutant selection strength, mutation timing and cell pushing strength respectively. Inferred parameter value is indicated by the vertical dashed line along the diagonal panels. 95% credible regions lie within the shaded region in the diagonal panels. Where no shaded region is given, this interval was the entire parameter range. (c) All analysed simulated sub-clonal mixing patterns (grey points) with CMFPT value of the BaseScope sub-sample (star) and posterior samples (green points). (d) Best-fit simulated sub-clonal pattern and parameters representing the most abundant parameter combination within the posterior distribution. (TIF) [file pcbi.1010952.s025.tif]

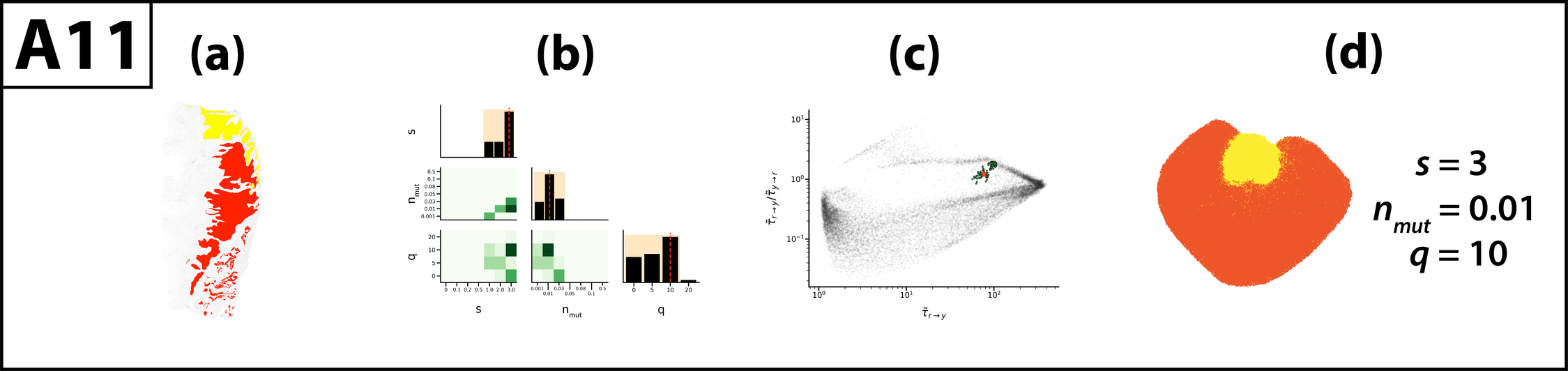

Supplement: S26 Fig — (a) Sub-sample of sample A11. (b) Marginal posterior distributions of model parameters s, nmut, and q, representing mutant selection strength, mutation timing and cell pushing strength respectively. Inferred parameter value is indicated by the vertical dashed line along the diagonal panels. 95% credible regions lie within the shaded region in the diagonal panels. Where no shaded region is given, this interval was the entire parameter range. (c) All analysed simulated sub-clonal mixing patterns (grey points) with CMFPT value of the BaseScope sub-sample (star) and posterior samples (green points). (d) Best-fit simulated sub-clonal pattern and parameters representing the most abundant parameter combination within the posterior distribution. (TIF) [file pcbi.1010952.s026.tif]

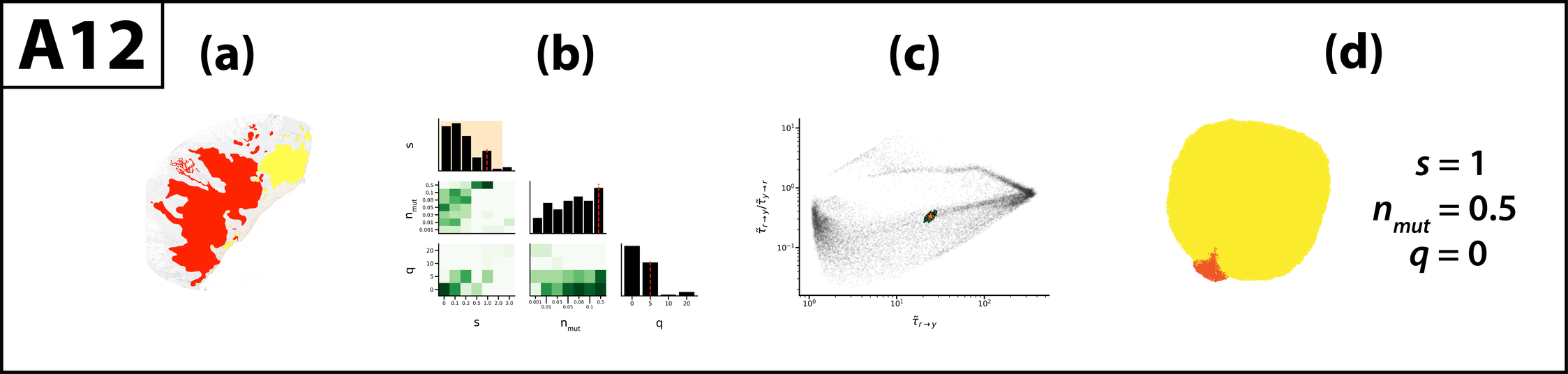

Supplement: S27 Fig — (a) Sub-sample of sample A12. (b) Marginal posterior distributions of model parameters s, nmut, and q, representing mutant selection strength, mutation timing and cell pushing strength respectively. Inferred parameter value is indicated by the vertical dashed line along the diagonal panels. 95% credible regions lie within the shaded region in the diagonal panels. Where no shaded region is given, this interval was the entire parameter range. (c) All analysed simulated sub-clonal mixing patterns (grey points) with CMFPT value of the BaseScope sub-sample (star) and posterior samples (green points). (d) Best-fit simulated sub-clonal pattern and parameters representing the most abundant parameter combination within the posterior distribution. (TIF) [file pcbi.1010952.s027.tif]

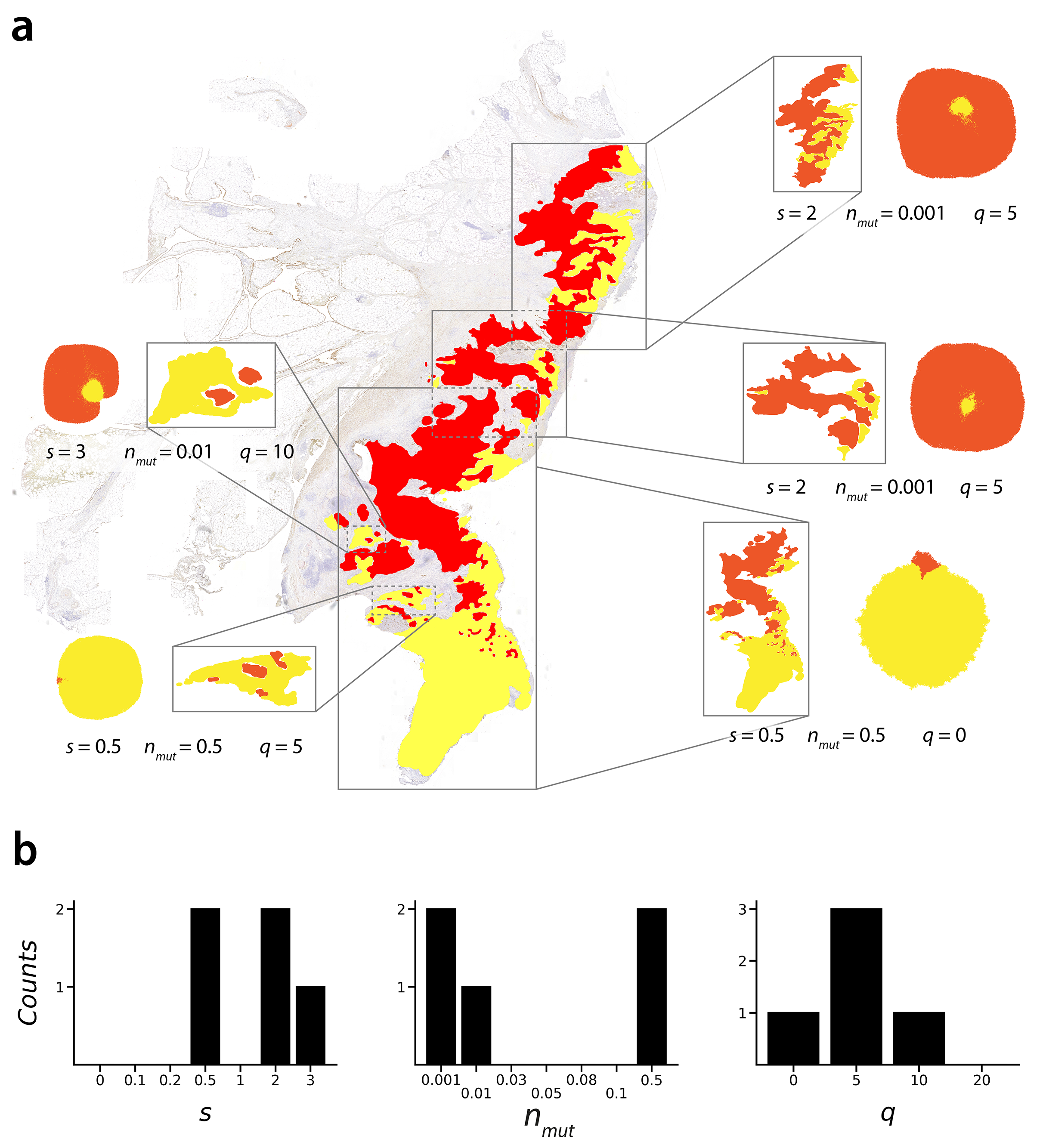

Supplement: S28 Fig — (a) Best-fit simulated sub-clonal pattern shown next to each sub-section with corresponding model parameters. (b) Marginal distributions of inferred model parameters across all sub-sections. (TIF) [file pcbi.1010952.s028.tif]

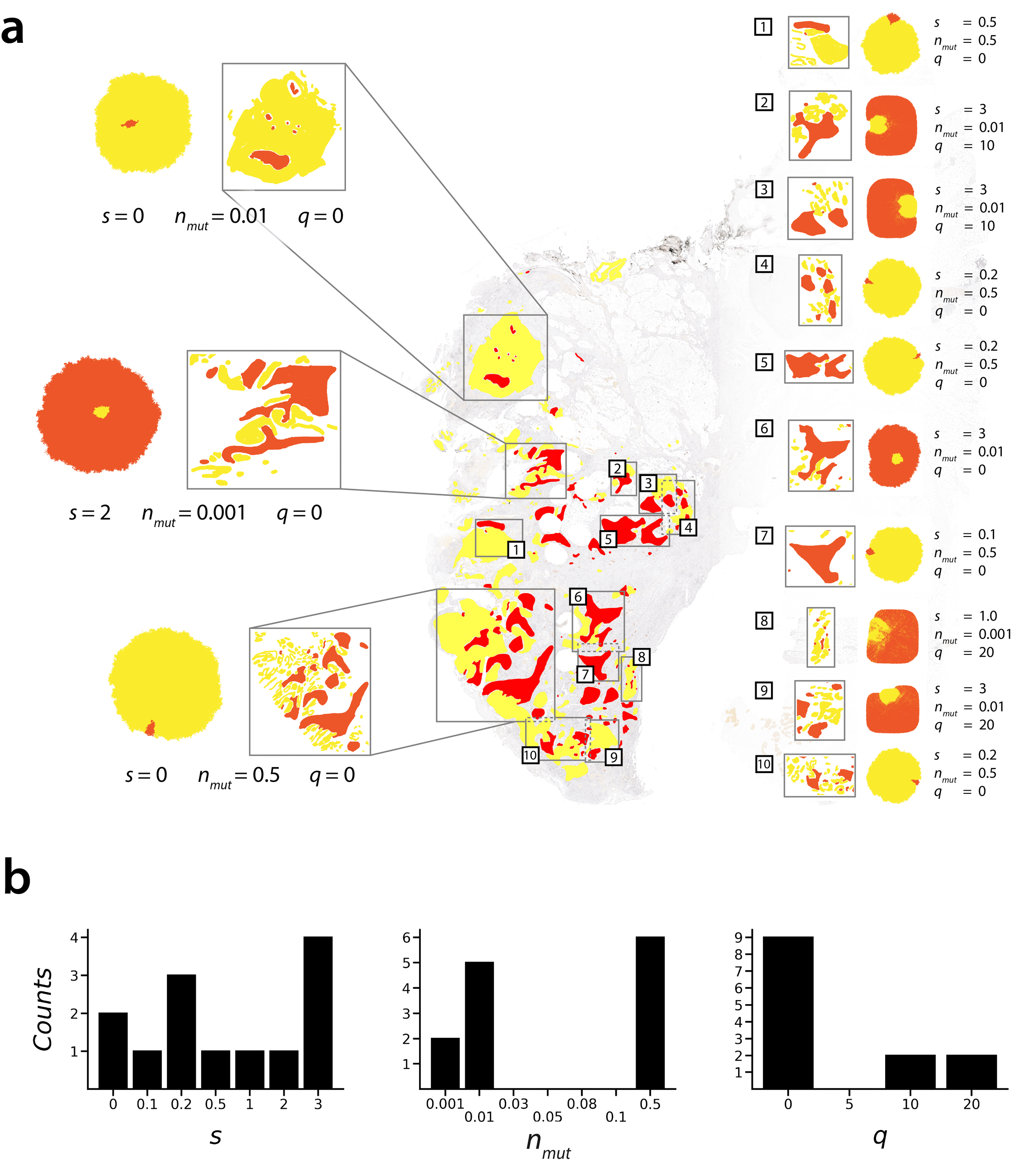

Supplement: S29 Fig — (a) Best-fit simulated sub-clonal pattern shown next to each sub-section with corresponding model parameters. (b) Marginal distributions of inferred model parameters across all sub-sections. (TIF) [file pcbi.1010952.s029.tif]

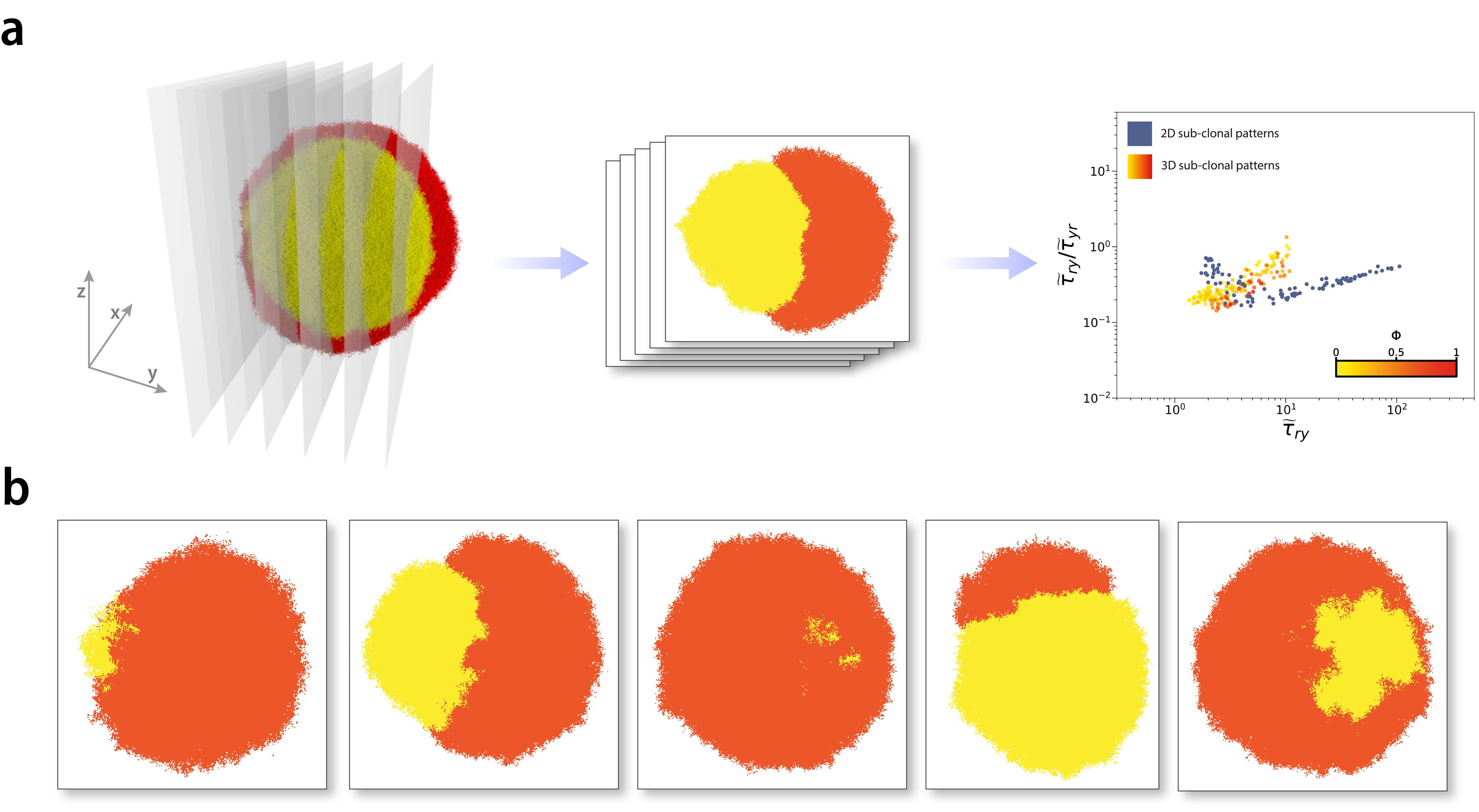

Supplement: S30 Fig — (a) 3D simulated tumour with (s, nmut, q) = (1, 0.01, 0). 2D slices are extracted from 3D tumour, with the observed 2D spatial patterns analysed using fractal analysis. In total, approximately 10 3D tumours were simulated for each of the following combination of parameters; (s, nmut, q) = (0, 0.001, 0); (0, 0.1, 0); (0.5, 0.001, 0); (0.5, 0.5, 0); (1, 0.001, 0); (1, 0.1, 0); (1, 0.1, 10); (3, 0.001, 0); (3, 0.1, 0); (3, 0.1, 20); (3, 0.5, 0) (b) Examples of 2D slices obtained from the 3D tumour in (a). (TIF) [file pcbi.1010952.s030.tif]

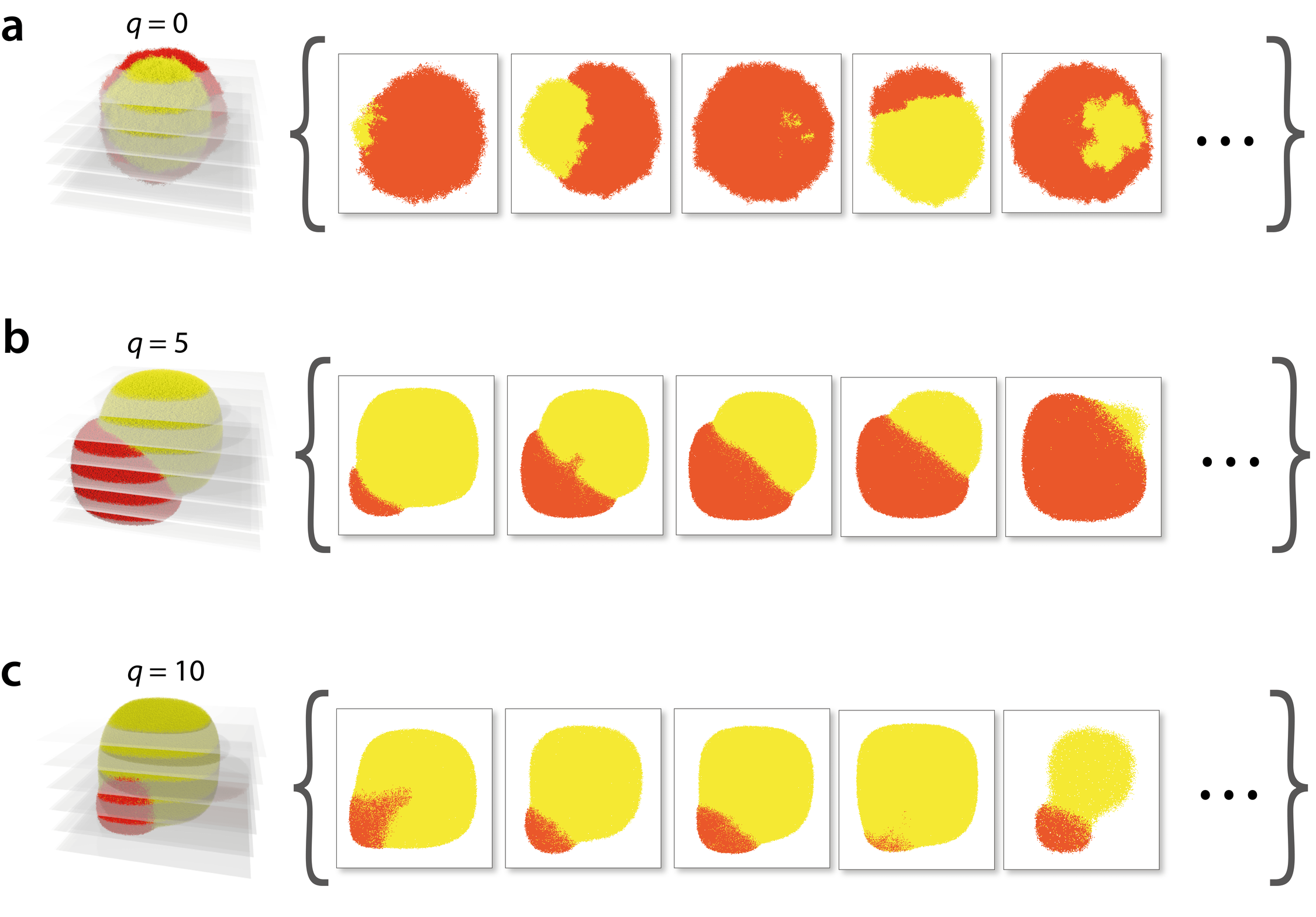

Supplement: S31 Fig — 2D sampling of 3D simulated tumours with model parameters s = 1, nmut = 0.01 (a) q = 0, (b) q = 5 and (c) q = 10. Samples are obtained by sweeping through the 3D tumour along three orthogonal axes (one direction of sampling depicted in 3D images on left of each sub-figure). (TIF) [file pcbi.1010952.s031.tif]

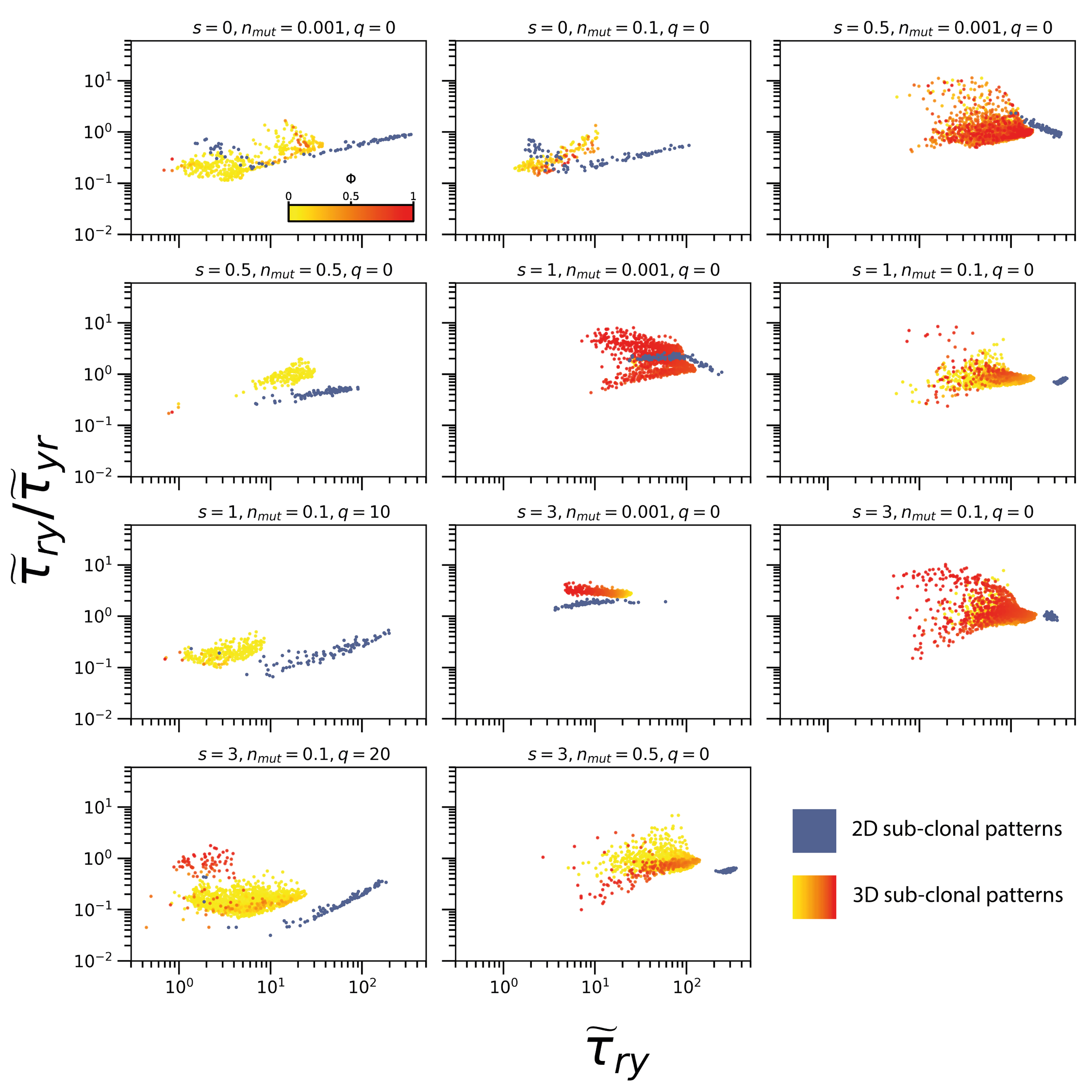

Supplement: S32 Fig — CMFPT analysis of sub-clonal patterns generated using 2D and 3D simulations for a range of sub-clonal parameters. CMFPT measurements are plotted in the (τ˜ry,τ˜ry/τ˜yr) phase space) with measurements of patterns derived from 3D tumours coloured according to their ratio of mutant to WT cell numbers, ϕ. Measurements of sub-clonal patterns generated with the corresponding 2D system are coloured blue. (TIF) [file pcbi.1010952.s032.tif]
