# Supplementary material for: First passage time analysis of spatial mutation patterns reveals sub-clonal evolutionary dynamics in colorectal cancer
Source: PLoS Comput Biol. 2023 Mar 13;19(3):e1010952. doi: 10.1371/journal.pcbi.1010952 (PMC10035892; doi:10.1371/journal.pcbi.1010952)
Supplement: Attachment — Submitted filename: response_to_reviewers.pdf [file pcbi.1010952.s035.pdf]

All line references in this response letter are made with respect to the marked-up copy of the revised manuscript (revised_manuscript_marked_up.pdf)

Reviewer #1: *The authors present an interesting manuscript that addresses the need for methods to analyze dynamic data for which there is only a single timepoint available. The authors utilize a method previously employed in other fields and consider its application to the study of cancer evolution. By using computational simulations (for which the ground truth is well known) and clinical data, the authors provide evidence of the usefulness of their proposed method. While I think that this research is significant and worthy of publication I found some issues (some more serious than others). I don't expect to have these points addressed in the research per self but at least addressed in the discussion in ways that ensure that the reader is aware of the limitations of the work which, otherwise, remains novel and interesting.*

The finding that pushing has a huge impact the topology seems relatively trivial if one considers that in this ABM a relatively similar effect could be obtained by adding cell motility.

The model is 2D but I think that while reality is 3D, much can be gained from 2D sections that reflect real evolutionary patterns. A different matter is the impact of the normal cells and the physical environment which is neglected by this work (admittedly and upfront). Contrary to what the authors claim this is no minor neglect, it is clear it will impact evolutionary dynamics (interactions between normal and tumor is not just about competition for resources and there are plenty of non cell-autonomous interactions) and while not easy, if they look in the literature they will find plenty of examples that demonstrate that this is possible and frequently done.

We thank the reviewer for their comments and careful consideration of our manuscript. We agree that a similar effect could be obtained by adding cell motility, albeit with some possible change to the typical cluster size of sub-clones in a motility model, compared to our pushing model. Some important mechanics such as short-range cell motility, tumour-stroma interactions and the significance of the physical environment are not included in our model to maintain the simplicity of our analysis. Our main aim of this paper was, however, to introduce a new method to recover evolutionary parameters from spatial patterns and demonstrate the efficacy of the CMFPT framework as a promising new route to achieve this in patterns generated by a simple and generic model. Following this, we then wanted to explore possible applications of this approach to study sub-clonal dynamics in human cancers. We now acknowledge these limitations of our model here and have added further discussion to the revised manuscript, between lines 565-599, where we address these shortcomings of the model. In addition, we have added lines 682-696 in the revised manuscript where we explain and give justification of the chosen ranges for the model parameters used in this study.

Whilst beyond the scope of this study, we were interested to know more about the impact of our choice to perform spatial modelling in 2D. In a new section of the paper, between lines 370-421, we extended our model from 2D to 3D and simulated tumours over a range of sub-clonal parameters. Our simulations highlighted the significant increase in complexity of the observed spatial patterns when obtained from 3D tumours, compared to 2D tumours, and that explicit 2D simulations produce only a subset of the possible patterns observable in 2D samples of the corresponding 3D system. Importantly, however, these results are in line with the reviewer's intuition, suggesting that the 2D model patterns provide a faithful, albeit incomplete, representation of the same dynamics in the 3D systems. We discuss these factors and the need for a future in-depth studies of 3D spatial patterns and the influence of tissue sampling on the inferred sub-clonal dynamics.

Furthermore, while it is mentioned that inter patient variability could be quite high, it would seem to this reviewer that this variability might be a key factor that could explain different evolutionary trajectories between patients with similar genetic (original) makeup.

We agree that interpatient variability is likely to be a key factor explaining the different dynamics inferred using our method, along with possible variability of the tissue sampling process (the latter point we also acknowledge in the revised manuscript in lines 460-462). To address the former, we have added some further discussion in lines 552-563 in the revised manuscript to acknowledge the impact of inter-patient variability in our samples.

Please, in the revision embed the figures with the manuscript, it makes for much easier reading! Also, for some reason the quality of the main figures (but not of the supplementary ones) is very poor and the axis and legends are very hard to read.

We thank the reviewer for taking note of this. We have taken extra care with the formatting of the main figures and have embedded them in the revised manuscript.

Reviewer #2: *There are some interesting ideas and a lot of work in this manuscript, combining theory and analysis of experimental data. However it does not convincingly shows any usefulness of the developed method. On the one hand, it states that this method is more detailed than another already existing method, but the manuscript does not show enough comparisons with existing methods, and one could think of other more straightforward methods which would similarly convey the spatial arrangement in simpler observables. On the other hand, there are several aspects of the method that are questionable, in particular the question of the number of cells considered, and that the information on the proportion of mutant area is not used; on top of the acknowledged issue of the 2 dimensional representation of the 3 dimensional process. For all these reasons, I recommend rejection of this manuscript despite some other qualities.*

Below, more about the 2 main points; and then a list of smaller comments.

** Main point 1: not enough comparisons with existing methods*

One comparison not directly made is with the previous method using Shannon's entropy. In the paragraph starting around line 386, "These analyses demonstrate how the CMFPT provides a more detailed description of the patterns of mutant sub-clonal mixing than the spatial Shannon's entropy measure that was previously used to analyze the BaseScope patterns" : there is actually no direct comparison with previous results. "Whilst the patterns produced under a surface growth model and one with cell pushing were qualitatively similar when analysed using Shannon's entropy, such dynamics were measurable when analysing the mixing patterns at multiple spatial scales using the CMFPT". If one looks at figure S6 where triangles represent values for experimental images, and figure S5 which shows the simulated points for each panel a different q value (large q values are strong cell pushing, q=0 is surface growth model), it is not clear that the experimentally observed regions of the taury, taury/tauyr space belongs more to a q value than another. Figure 5f shows the comparison between expected and inferred values of the various parameters for simulated data (much easier to compare than experimental data of course), and, while there is some signal, there is a relatively high error rate.

We thank the reviewer for their careful assessment of our manuscript, and agree that the data obtained using spatial analysis approaches is noisy and that patterns with different underlying dynamics can lead to overlapping clusters of CMFPT values in the (Tr->y, Tr->y / Ty->r) phase space. This is the underlying reason for the relatively high error rates we report in Figure 5f. We attempt to address the signal-to-noise ratio using our Bayesian style grid-search approach to inference where, for each experimental pattern, we find the *n* simulated patterns which are closest in the phase space. By doing so we hope to draw out the signal from the noise.

We acknowledge the reviewer's concerns about the lack of comparison to methods such as Shannon's entropy. To address this, we have performed new analysis of the patterns generated in Figure 2, and the simulated tumour patterns, using the same Shannon's entropy approach previously used to analyse the same experimental data of this work (Baker, AM., Huang, W., Wang, XM.M. et al. *Nat Commun*, 2017. https://doi.org/10.1038/s41467-017-02295-5). We present our new results in Supplemental figures 1 and 2, which show that CMFPT improves upon Shannon's entropy approach in analysing spatial heterogeneity. We discuss these analyses in lines 120-134 of the revised manuscript. Especially when applied to the simulated tumour patterns, we find that using information on the Shannon's entropy and mutant ratio (phi) of a pattern is only marginally better than simply matching images based on their mutant ratio alone. Furthermore, our exploratory 3D simulations, which are also newly included in the revised manuscript, demonstrate that mutant ratio is likely a bad predictor of the underlying dynamics when comparing experimental data to the 2D simulation model.

In the discussion, in the paragraphs between lines 426 and 462, the authors actually compare with another more sophisticated method, and while for some data there is some qualitative agreement between this manuscript analysis and this other study, there are disagreements with other data. So it is not very conclusive.

As the reviewer points out, the study to which we compared a small number of our samples utilised an enormous amount of data in the form of multi-region whole genome sequencing data. We agree with the sentiment of the reviewer that the comparison is not very conclusive, yet we believe the comparison of our more novel approach of studying spatial mutation patterns to a more standard technique adds value to our discussion. We have altered the wording of the relevant section in the discussion, between lines 505-508, to acknowledge the difficulty of comparing the two methods and that our method is not more informative than standard sequencing approaches.

Besides, the main point is to represent with 2 observables (the rescaled tau r->y and the ratio of the rescaled tau r->y / tau y->r) the spatial distribution (homogeneous domains vs. more diffuse). It is done using a complicated metrics, the class mean first passage times (CMPFT). I have nothing a priori about calculating MFPT, but I don't really see how that conveys more information than metrics such as a mean shortest distance, which would be much easier to compute, and also easier to check its validity for known patterns, such as those generated in figure 2. It seems complex for no real gain.

We have performed new analysis of the spatial patterns generated in Figure 2, and simulated tumour patterns, using mean shortest distance. We agree with the reviewer and show that the results using mean shortest distance are similar to those derived using the CMFPT (Supplemental figures 3 and 4 in the revised manuscript). We argue, however, that the CMFPT conveys slightly more information about the pattern in question, due to the process of finding the distribution of distances from cell type A to B using a stochastic process, compared to the deterministic process of finding the shortest distance (i.e. the approach of the mean shortest distance metric). This means that the CMFPT enables us to obtain a distribution of transition times for every starting node in the network, unlike the mean shortest distance, and therefore conveys more information about the neighbourhood of the starting cell. We have included some analysis and comparison of the mean shortest distance approach in the revised manuscript between lines 136-149.

** Main point 2: questionable aspects of the methods*

The authors do acknowledge that the real process is 3-dimensional, while their simulations that they use as a model, to infer to which parameters the observables correspond to, is 2-dimensional. A 2-D slide of a 3-D process is indeed quite different from the outcome of a 2D process. To give an example, let's imagine 2 sheets, one with only WT, the other with only mutants, so a very segregated process. If both sheets are wrinkled, and there are on top of each other, an horizontal plane cutting through both sheets will get some of the bottom sheet, then some of the top sheet, then the bottom, then the top, and so on, giving the impression of a much more diffuse process. At least on this point the authors are aware of the potential limitation.

The reviewer rightly points out this important limitation of the study. We are certainly aware of this limitation, however to fully understand the implications of a 3D process was beyond the scope of this study. As such, we chose to simulate in 2D in order to develop the spatial analysis framework in a simplified setting, and to demonstrate that the CMFPT can be used to recover dynamic quantities of a system using only the resulting spatial information. We are of course also very interested in the difference between explicit 2D and 3D simulations, and so have performed some exploratory simulations of explicit 3D tumours, using the same rules and parameters as the 2D analogue. We discuss this new work in lines 370-421 (and Supplemental figures 29, 30 & 31) of the revised manuscript. From these 3D tumours we sampled 2D slices of cells, and performed some basic analyses of these images. One of our observations was exactly what the reviewer describes, where some mixing of WT and mutant cells is observed for certain 2D slices, even for boundary driven tumours (q=0) where no cell-cell displacement occurs. More generally, these simulations highlight the significant increase in complexity of the observed spatial patterns when obtained from 3D tumours, compared to 2D tumours, and that explicit 2D simulations produce only a subset of the possible patterns observable in 2D samples of the corresponding 3D system. We discuss these factors and the need for a future in-depth studies of 3D spatial patterns and the influence of tissue sampling on the inferred sub-clonal dynamics.

Around line 330, it is acknowledged that scale can be important, as when discussing the case with analysis of subparts of an image, it is stated that "(...) the perceived cell pushing strength may be most affected by the sub-sampling strategy employed here, as even highly mixed patterns will begin to appear segregated when viewed with a higher magnification". However nowhere there is any discussion of whether the right scale is used. The simulations are done for clusters stopping at Nmax=10^5 cells. Does it correspond to the right order of magnitude in the imaged tumors? How sensitive are the estimates to perform the simulations with different Nmax? Also, the experimental images are subsampled to 3000 pixels (images of 3000x3000?) (paragraph starting line 342). The simulations run for Nmax=10^5 in 2D, thus typically a final size of 300 cells x 300 cells, thus smaller than the subsampled images of the tumors. That the scales are not matching may be an issue for parameter estimation, and this is not at all explored.

We thank the reviewer for this comment. The number of cells contained within each of the experimental samples is of the same order as Nmax, and so in this regard there is parity between the simulated and experimental images (experimental images are very approximately (8000μm x 8000μm), and assuming tumour cells of the order of (10μm x 10μm) this gives, considering the amount of white space and non-tumour cells in each image, a very generous upper bound of 6.4x10^5 cells per image, to first approximation). The resolution (i.e. number of pixels) of the experimental images, however, is far higher than Nmax. We downsample the experimental images to 3000x3000 pixels, which is still larger than Nmax, however one strength of the CMFPT approach is that we normalise each measurement to one derived under a null model of uniform random population mixing. This means that it is possible to compare images which are different sizes, so that simulated and experimental images may be compared. We have added some discussion about this topic in lines 252-257 of the revised manuscript.

In the section starting around line 587, what is explained is a method finding the simulations best matched with the experimental patterns, in regards to tau_rr, tau_ry, tau_yr, tau_yy. Why not using phi, the proportion of a given color, as an additional info? It's very easy to compute, and it is concerning than in figure 6, triangles representing experimental images have sometimes very different color (representing phi) than the simulated points to which they seem close to for the CMFPT values.

Information about phi does convey information about the dynamics and is very easy to compute, as the reviewer points out. Indeed, in previous analyses of the experimental colorectal cancer samples, which utilised a simpler Shannon's

entropy measure, data about phi was included in the inference framework. We chose not to include this information in our analysis for two main reasons. First, and more trivially, we wished to explore the efficacy of the CMFPT measure alone in quantifying the pattern topologies and linking these to the underlying dynamics. Second, more importantly, whilst phi is rather strongly coupled to the underlying dynamics in a 2D system, it is much less informative about the dynamics in 2D samples of 3D systems. This claim is supported by our exploratory simulations using an explicit 3D model, which we have now included in the revised manuscript, which demonstrate the wide range of possible phi values for different 2D samples of the same 3D tumour. Including information about phi would certainly have improved the inference sensitivity analysis we presented in Fig 5f, where we compared 2D images to 2D images, however this is not the case when we apply our analysis to real tumour data, which are 2D samples derived from 3D tumours. Despite this, phi still carries information about the underlying dynamics, and part of an extensive study using 3D simulations in the future should be focussed on better understanding the range of phi values for different 2D sample orientations, and the probability of measuring a certain phi given the underlying dynamics. Then, this information might be incorporated into inference frameworks. We have acknowledged this issue in lines 525-533 and 710-717 of the revised manuscript.

** smaller comments (more or less in the order they appear in the manuscript):*

- the value of Nmax and other parameters should be in the legends of the main text, not to be dug up somewhere in the SI

We thank the reviewer for highlighting this. We agree and have added parameter values to the legends of Figs 3, 4, S2 & S4-S8.

- line 109: should be 0 < phi < 1, not the <= and >=, as the quantities are not defined for phi=0 and phi=1.

We have changed this in line 104 of the revised manuscript.

- I found it a bit confusing to use tmut as a name for the parameters representing the proportion of Nmax, which when reached, makes the simulation introduce a mutant. It is not directly a time, as the number of new cells in a given unit of time scales as the number of existing cells.

We agree that this is a bad choice of notation, and have changed t_{mut} to n_{mut} throughout the revised manuscript. We retain our language of sub-clone emergence “time” in the manuscript, but have added some explanation in lines 159-168 of the revised manuscript addressing the correct interpretation of this parameter.

- Around line 166 "Due to the coupled effects of s and t mut on the final class ratio, tumors with early arising, weakly selected mutant sub-clones (Fig 4, small blue points), and those with later arising, strongly selected sub-clones (Fig 4, large red points) share similar values of phi (shown by colors in Fig 4a-d, inset). Interestingly, despite having a similar mutant frequency, phi, the CMFPT is sensitive to the topological differences between patterns generated in these two contrasting scenarios. Tumors with the earliest arising sub-clones occupy a unique area of the phase space for all cell pushing strengths." Figure 4 there are clearly point with warmer colors (larger s) and larger size (larger tmut) colocalized with points of colder colors and smaller size (smaller s, smaller tmut). So it is actually not really the case that the CMFPT is able to contrast these 2 scenarios.

The reviewer fairly highlights this mischaracterisation of our method. It is true that different dynamical regimes can lead to similar spatial patterns, which lead to similar CMFPT values which may be difficult to distinguish. We have changed lines 192-199 in the revised manuscript to clarify our commentary and avoid overselling the efficacy of the method.

- All the discussion around page 11 about the non-tumorous tissue. Removing it and selecting subparts by hand may introduce bias. Actually it is also removing additional information that could be used in a more sophisticated model.

We agree with the reviewer, and note that, when applying our analysis to real human data, the approach of removing the non-tumorous tissue and selecting subparts was driven by the shortcomings of the computational model. It is certainly the case that spatial information contained within the topology of the non-tumour population would offer more information about the dynamics of the tumour populations, and this could be exploited with more sophisticated models in future work. We have acknowledged these points in lines 535-550 of the revised manuscript.

- Could q be related to real mechanical properties of the samples?

We believe so, yes. For example, *q* could relate to the local cell density and physical stresses within the tumour tissue. These properties will vary between patients, and perhaps even across different areas of the same tissue. We acknowledged and discussed this briefly in lines 361-368 of the revised manuscript.

- line 551 : stochastic birth : a stochastic birth rate may not be ideal for cells (this leads to an exponential distribution of

duration between replications, with often very short times, whereas cells require a minimum time to be able to divide). That's an approximation often made, but that could be briefly discussed.

We thank the reviewer for pointing this out, and agree that it should be addressed in the text. Our decision to model cell replication in this manner was motivated by computational efficiency, model simplicity and avoiding lattice artefacts. We have added a comment in lines 661-663 of the revised manuscript to acknowledge this model assumption.

- equation (2) : (1+s)^k with k in {0,1} : why this definition? Why the need of putting k rather than just 1?

This choice of definition was made so as to make the notation as general as possible, since the simulation model in principle can simulate multiple driver mutations. Since we only every simulate 0 or 1 driver mutations in this study, however, we agree that this notation is clumsy and have changed Eq. 2 in the revised manuscript.

- around line 566 : note that this models gives more death when there is more replication. Why not, but this could be discussed as it is not self evident.

We have added some more text in the revised manuscript between lines 666-668 acknowledging this feature of the model and offering some biological justification for this choice.

- figure 4 : the color of the small points cannot be seen.

We thank the reviewer for highlighting this issue and have re-plotted Fig 4 in the revised manuscript.

- figure S1a : some scale bars are seen : which size do they correspond to?

Scale bars represent 2000 microns. We have added this information to the figure caption for Fig S9.

Reviewer #3*: I believe this is a potentially interesting contribution to our understanding of early cancer development and emergence of different subclones in space. However, I have some concerns related to the modelling choices, its impact, and possible overselling of the clinical impact.*

1 Modelling choices
1A If I understand it correctly, there is no interaction of cells of different types assumed, beyond the cell pushing. It seems to be a strange assumption to me (the interactions more likely happen than do not happen in a situation where cells need nutrients to proliferate and survive and where space/resources are limited). The fact that we need cell pushing for division implies the space is limited, thus at least I would expect some sort of competition for space/resources instead of only pushing another cell when dividing. In that sense, I was surprised that this assumption was not listed among limitations of the modelling framework

We thank the reviewer for their comments on our manuscript and agree that our simulation model is simple and does not account for many of the biological and ecological process which are important in cancer evolution. Our choice of model design was motivated by our desire to develop a simple model with parameters which might each have different influences on the spatial distribution of the resulting mutated sub-clone. One could conceive of many other more complex models, but our main message of this study was that we were able to use the CMFPT measure and spatial information alone to recover parameters of a generative model, provide some useful applications to spatial tumour data. We fully agree with the reviewer, and it is fair to our audience to know about our rationale of model choice and the consequent limitations. We have added some more discussion in the revised manuscript between lines 565-599 where we address the shortcomings of our simulation model and some of the important biological processes for which our model does not account.

1B How does the fitness advantage relate to the common assumption of resistance cost, i.e. that therapy resistant cells proliferate slower in absence of treatment? And can there be a temporal fitness disadvantage, followed by a fitness advantage, for example? For example, can the fitness advantage happen only in a long-term once the resistant subclone outcompetes the sensitive one more effectively? And can you imagine the fitness advantage having a form of competitive advantage at all? Would it make a difference?

We agree with reviewer that the resistance mechanism is complex, and that comparing scenarios with or without fitness cost is a very reasonable expectation. However, in our patient samples, the mutated genotype is caused by simple point mutations (KRAS mutations) pre-existing before targeted therapy. In addition, most of our patient samples are pre-treatment, and so if there is a fitness cost in absence of treatment in these samples, it is unlikely that we would observe any cases of high frequency of resistant cells.

From a modelling perspective, we are not concerned specifically with resistance per se, but focus more on quantifying the spatial heterogeneity of different cell types through the CMFPT method. These cell types can be resistant versus sensitive genotypes, or genotypes with different mutations, or any other cell types which can be distinguished with some markers spatially. We have edited the language used throughout the manuscript to avoid implying any assumed resistance to the reader, and specifically we have made edits between lines 2-8 in the manuscript introduction to address this issue. On the topic of temporal fitness disadvantage, followed by neutral or fitness advantage: these dynamics would surely affect the resulting spatial pattern, but we believe our framework in its current form is not powered to detect such dynamics.

1C The cell pushing, especially adopted to a 2D lattice model, sounds very unnatural. In reality, the space is continuous and three-dimensional. Could you motivate why having the extension of Waclaw et al.'s algorithm makes your Agent-Based simulation more realistic and not less realistic? Isn't cell pushing you implemented in your 2D lattice model the main cause of the surprising results for patient C537? How could you validate whether this is the case?

Lattice models are very commonly used in computational biology to model expanding tumours. These are of course an approximation of real systems, where space is continuous, and lattices can introduce lattice artefacts, however other analysis of our model (using a different metric to CMFPT and exploring the shape of the population boundaries specifically) suggested that our choice of lattice structure does not significantly impact our conclusions about the dynamics. The alternative of modelling off-lattice introduces significantly more complexity into simulations and might obscure our message in the paper which is about the CMFPT framework. Moreover, off-lattice simulations are far more computationally expensive, and we would be restricted to simulating tumours orders of magnitude smaller than those possible with our lattice model.

We chose to adopt the pushing algorithm of Waclaw *et al.* since it is slightly more realistic than simple straight-line pushing. Straight-line pushing algorithms may simply involve finding the nearest empty lattice point to the dividing (and therefore pushing) cell and pushing all cells that fall along the straight line connecting these two points. The algorithm of Waclaw *et al.*, however, involves constructing a chain of cells, beginning with the dividing cell, by finding

the nearest empty lattice point with respect to the cell at the end of the chain each time, and adding the neighbouring cell in that direction to the end of the chain. The Waclaw *et al.* algorithm therefore reflects, to some extent, the local tissue density at each point along the chain of cells being pushed and ensures that cells move down pressure gradients all along the chain.

Regarding the surprising result for C537: we believe a more likely reason for the surprising result is the difference in the data which were used in our study, and the study of Heide *et al*. Their computational model is quite similar to ours, and they employ a straight-line pushing algorithm. As we have later ascertained from our 3D spatial modelling results, the impact of spatial sampling of the tissue itself, and stochasticity associated with this process, can significantly alter the conclusions we make using our approach of analysing the sub-population boundaries. Many more simulations would be required to quantify the likelihood of sampling 2D cell patterns like those observed in our samples from C537 (A7, A10 & A11) from a 3D tumour harbouring a neutral sub-clone (as the model of Heide *et al.* suggested for this patient), which would offer more insight into the sub-clonal dynamics in this patient.

1D I think exclusion or inclusion of other than cancer cells in the modelling should be motivated by type of cancer, its stage, and considered treatment. Therefore, I was rather surprised to not see immune cells in your model as you have an early stage of cancer with known strong involvement of immune cells. More discussion on this would be appreciated. (This however seems to be less critical than no interaction among different subclones.)

Similar to our response the reviewer's point 1A above, our goal was to demonstrate the efficacy of the CMFPT analysis to recover and estimate the underlying dynamics of a system based on the spatial information alone. The CMFPT analysis can be applied to quantify the spatial heterogeneity of any different cell types, e.g. different types of tumour cells, resistant versus non-resistant cells, and so on. Here, we demonstrate it with a simple setup of mutated and non-mutated cells, because in our patient samples those are the only cell types identified using the RNA BaseScope technique. Despite this, we agree with the reviewer that the role of immune cells is important especially in colorectal cancer. Our computational model could certainly be tailored more towards any specific cancer type and stage if applying to a different experimental data setup e.g. with more spatial information of immune cells. In addition to toning down the statements about the clinical impact of our work, which we hope should now be proportionate to the scope of the simulation model, we have addressed this aspect of the model and other model assumptions in a new section in the discussion between lines 565-599.

1E To perform inference of mutant replicative advantage (s), mutation timing (tmut) and cell pushing strength (q) for each BaseScope sub-sampled region a grid-search was used. I do not understand why optimization was not used instead and how the specifics of the grid search were chosen (for example q=0, 5, 10 or 20 - why not other values? Or did I miss it?). Could you elucidate on this?

We chose to perform a grid search because both generating the simulated tumour patterns and analysing them with CMFPT is computationally rather slow. One could in principle implement a Markov chain Monte Carlo method, however we chose simply to analyse a large pre-existing set of tumour patterns with the CMFPT and perform a grid search to find which best described the experimental patterns. Our chosen range of q values was to cover a range of growth modes, from surface growth (q=0) to more faster growing tumours with increasingly stronger pushing strengths. Different choice of q values will lead to some quantitative difference but not qualitative difference in our conclusions. We have added a paragraph between lines 682-696 in the revised manuscript where we explain and justify our choices of parameter values used in this study.

2 Impact of the modelling choices
2A My belief is that at best, you provide a plausible hypothesis for emergence of subclones in space. I wonder what are other options, and how critical the choices you made (see part 1) are for your observation. What about the cell pushing being replaced by placing offspring to the nearest free space and including rather standard competition among different cell types, for example? Would that lead to the same outcomes? I have no intuition regarding this.

We would expect other options, such as the "short range motility" dynamics the reviewer suggests, could have similar effects on the extent of wild-type and sub-clonal population mixing as our pushing mechanics. One important difference with the short range motility mechanics, however, would be the absence of any displacement of the cells which lie between the dividing cell and the nearby empty lattice point. This might affect the size of sub-clonal clusters. With reference to our response to point 1A above, we acknowledge that many other model mechanics could be included or substituted into our model, and we address this in lines 565-599 of the discussion in the revised manuscript.

2B As the measurements are spatial, but include only small section of a tumor and 1 time point, many spatial models with enough parameters would fit this data well (enough). While I understand the choices you made, I question how could you validate your hypothesis better. Are there time-series data you could use, for example? Could discussion include thoughts on this, including alternative hypotheses?

Time-series data is very difficult and rare to obtain with this type of data, and we were not able to obtain time-series data for this study. Such data, coupled with appropriate computational modelling, would be very interesting and would present a unique opportunity to validate our model and study the effects of mutant sub-clonal growth in 2D/3D space. We have mentioned the lack of longitudinal sampling in the discussion of our revised manuscript, where we note that this is one area where approaches using generative computational models like our can be particularly effective in resolving/inferring temporal dynamics. We have also added some discussion in the revised manuscript, between lines 565-599, where we discuss other modelling choices and hypotheses that could be tested in future studies.

2C Page 18, line 333: "Validation of our inference method (Fig. 5f) would suggest that the majority of sub-regions across these samples were indeed consistent with weak cell pushing, however it is also plausible that stronger cell pushing was acting within these tumours, which led to the observed fragmented patterns of tumour cells, which in turn necessitated the sub-region analysis in these samples." Aside of discussion on how realistic the pushing is when converted to the 2D lattice model, how would you validate this alternative hypothesis?

In the revised manuscript, we include some preliminary analysis of sub-clonal patterns generated and sampled from a 3D spatial model. This work demonstrated that it is possible to observe intermixing of the wild-type and sub-clonal tumour populations, even in purely surface growth systems (i.e. q=0) if the 2D sample is acquired from the 3D tumour along certain planes. These results from our 3D simulations were intriguing, and further work in this direction could help to validate this hypothesis. Alternatively, they might confirm that weak cell pushing remains the most plausible dynamics in these samples, and that the random tissue sampling process is sufficient to explain the observed fragmentation in these particular patterns.

3 Possible overselling of clinical impact

The introduction starts with "Understanding the origins and effects of intra-tumour heterogeneity is a fundamental challenge in cancer research and is critical for managing therapy resistance and improving treatment outcomes."

3A As you consider only subclonal resistance (and not e.g. epigenetics, phenotypic switching) and as you assume that all resistance is pre-existent, your model would be valuable for cancers and treatment where this assumption is satisfied. Can you elaborate on how realistic these assumptions are, in general and for colorectal cancer?

In this study we aimed for a more general approach to quantify the spatial patterns of different cell types *i.e.* using random walk and first passage time to measure the clustering or mixing of cells. We apply this method to a population model of wild-type and mutated tumour cells mainly because this was the scenario in our patient samples. Point mutations (in the *KRAS, BRAF* and *PIK3CA* genes) were detected before treatment and spatial maps were constructed accordingly (Baker, AM., Huang, W., Wang, XM.M. et al. *Nat Commun*, 2017. https://doi.org/10.1038/s41467-017-02295-5). For this reason, in our application we modelled the "emergence" of the sub-clonal population. We are interested in characterising the effect of mutation emergence time (parameter t_{mut}, now n_{mut} in the revised manuscript) on the spatial pattern of the resulting clade. However, we fully agree with the reviewer that, in general, more complex mechanisms can lead to treatment resistance and we would need to extend our population model in applications to other scenarios. For example, our modelling approach could be adapted to study systems with phenotypic switching. The forward and backward switching rates would then become additional parameters of the population mode. We could model the phenotypic switching as an intrinsic rate or only triggered by treatment, and all these parameters could affect the patterns of sub-clonal clustering. We believe our work demonstrates that the CMFPT could be a promising new approach, adopted from the research field of complex systems, to analyse spatial heterogeneity in cancer. We have now explained these motivations and limitations more clearly to our readers.

3B Are you sure that understanding the specific mechanisms of resistance evolution help us to handle the worst cases, like metastatic cancers? From recent advances in evolutionary therapies in metastatic cancers I got an impression that it does not matter much how resistance occurs as long as we can address it strategically. Also, knowing how precisely the resistance emerged is I think impossible and likely not relevant for these advanced cancers. What am I missing?

We agree that determining precisely how sub-clones emerge is not possible, but we tried to apply our method to a set of real cancer tissue samples and explore if, within the framework of our computational modelling, we could correlate the timing and strength of sub-clones, and general cell pushing strength, to the resulting spatial patterns. One could perhaps, given a large enough data set, find that, for example, *KRAS* driver events tend to occur earlier than *BRAF* in colorectal cancer, but our application of CMFPT to this colorectal cancer data is just one application of this new method to cancer tissue data.

We appreciate the reviewer shared a perspective of handling resistance strategically rather than sourcing the origin of resistance mechanisms. This indeed would be an interesting topic to discuss in the field of cancer evolution. We would like to kindly point out that distinguishing different resistance mechanisms is not in the scope of our work. Instead, we

used the spatial data of wild-type and mutated tumour cells to show how to apply CMFPT as a novel approach to quantify the dynamics of cell populations using limited input information. We do not mean to argue that our method could or should replace other standard methods of studying cancer evolution.

One possible question connecting of our approach to metastatic cancers is whether comparing spatial patterns of tumour versus normal cells in different metastatic sites and the primary tumour can, to some extent, tell us whether metastasis is an event happening early or a continuous event happening over time. As shown in our results, the arising time of a type B cell in type A population will impact the spatial patterns of A and B. Similarly, we can imagine the time of the tumour cells arriving at the metastatic sites might lead to more similar or diverse spatial patterns of tumour versus normal cells across metastasis sites. This could be an interesting future application to explore, and we thank the reviewer for inspiring us to think in this direction.

In view of 3A and 3B, I would tune the statements on clinical implications (few in the paper) down.

We thank the reviewer and their comments about our claims of the clinical impact of our study, and have toned down the statements made in the revised manuscript. Specifically, we have changed the language used in the opening paragraph of the introduction, between lines 2-8, to tune down the statements on the clinical implications of our work remove specific discussion of evolution of resistance. We have made further changes to lines 608-610, to again reflect these comments of the reviewer.

4 Others

4A I believe one should understand a submission without the need to study other papers. Here I needed to read reference 20 to understand the scope. I would suggest to summarize earlier results, also including modelling, so that no reader needs to read other papers to understand your contributions.

We thank the reviewer and agree that our manuscript should be self-contained. We have added a short summary of reference 20 to the introduction in the revised manuscript between lines 25-32.

4B Page 23, last line: "In line with contemporary models of tumour evolution..." Are you sure that your results are really in line with all contemporary models of tumor evolution? Again, tuning this statement down would be better.

We agree that this statement should be toned down, and have changed the wording to “In line with some contemporary models…” and have cited some relevant papers. To tone down this statement further we have changed the wording of the following sentence, between lines 608-610, where we summarise the contribution of this study.

4C (minor) Page 20, line 403: Missing table number.

We have amended this missing table number.
