# Supplementary material for: First passage time analysis of spatial mutation patterns reveals sub-clonal evolutionary dynamics in colorectal cancer
Source: PLoS Comput Biol. 2023 Mar 13;19(3):e1010952. doi: 10.1371/journal.pcbi.1010952 (PMC10035892; doi:10.1371/journal.pcbi.1010952)
Supplement: Attachment — Submitted filename: comment.pdf [file pcbi.1010952.s036.pdf]

Column case

(here distances are in units of the total domain lenght. One fixed conversion factor to the results in the manuscript)

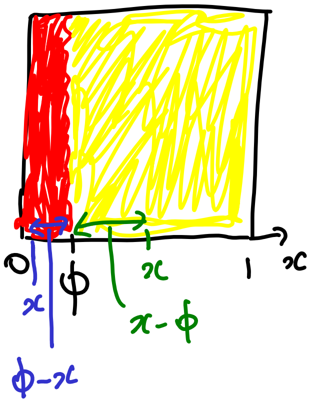

$$\tilde{d}_{ry} = \frac{1}{\phi}\int_0^{\phi} (\phi - x)\,dx = \frac{1}{\phi}\int_0^{\phi} u\,du = \frac{\phi^2}{2\phi} = \frac{\phi}{2}$$

$$\tilde{d}_{yr} = \frac{1}{1-\phi}\int_{\phi}^{1} (x - \phi)\,dx = \frac{1}{1-\phi}\int_0^{1-\phi} u\,du = \frac{(1-\phi)^2}{2(1-\phi)} = \frac{1-\phi}{2}$$

prediction for figure S3, right panel:

$$x = \tilde{d}_{ry} = \frac{\phi}{2}$$

$$y = \frac{\tilde{d}_{ry}}{\tilde{d}_{yr}} = \frac{\phi}{1-\phi} = \frac{2x}{1-2x}$$

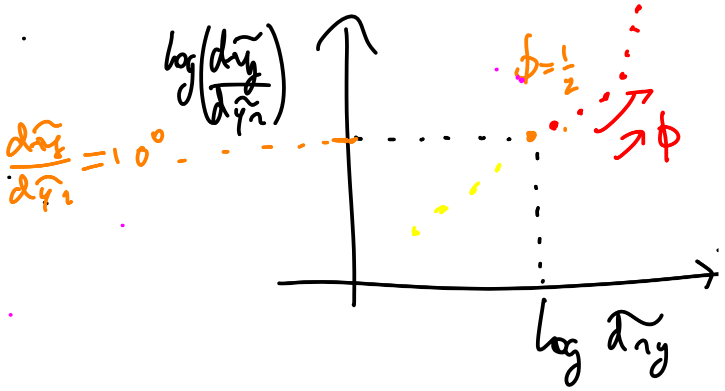

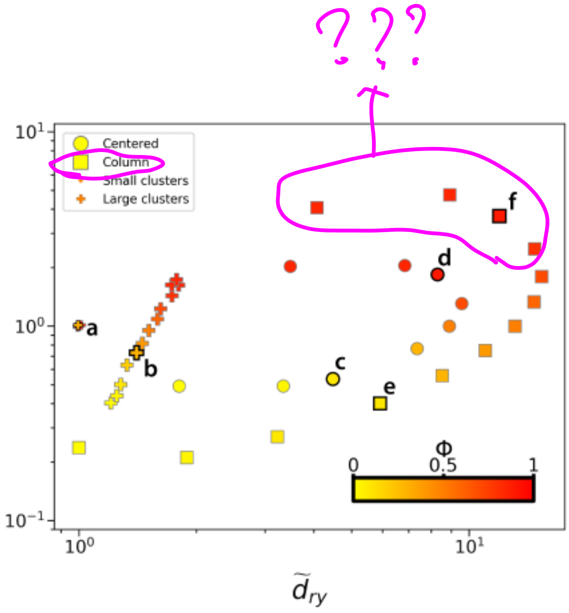

Small cluster case:

(assuming small red cluster in yellow domain, with density of clusters ↗ with φ)

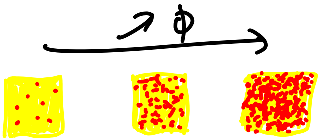

$\tilde{d}_{ry}$: mainly controlled by cluster size, thus ≈ constant.

$d\tilde{y}_r$: $> d\tilde{r}y$ … $\simeq d\tilde{r}y$

$\frac{d\tilde{r}y}{d\tilde{y}r} < 1$ … $\frac{d\tilde{r}y}{d\tilde{y}r} \simeq 1$

$\frac{d\tilde{y}}{d\tilde{x}}$ should be <1 for ϕ small →

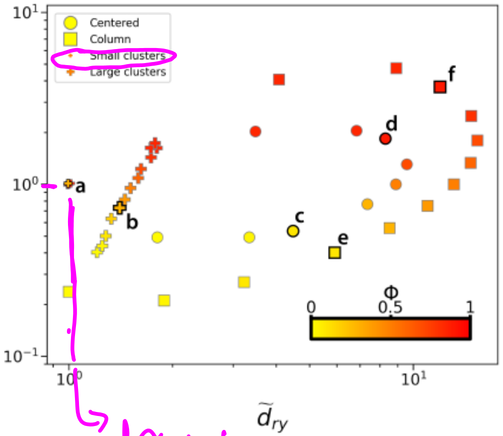

$d\tilde{y} \approx$ ct : OK.
