# Supplementary material for: First passage time analysis of spatial mutation patterns reveals sub-clonal evolutionary dynamics in colorectal cancer
Source: PLoS Comput Biol. 2023 Mar 13;19(3):e1010952. doi: 10.1371/journal.pcbi.1010952 (PMC10035892; doi:10.1371/journal.pcbi.1010952)
Supplement: Attachment — Submitted filename: author_response.pdf [file pcbi.1010952.s037.pdf]

December 9, 2022

Response to reviewer comments on "*First passage time analysis of spatial mutation patterns reveals sub-clonal evolutionary dynamics in colorectal cancer*"

We thank the reviewer for their response and careful analysis of our work. The reviewer raises concerns about the results presented in Figure S3, in which we plot mean shortest distance values computed for simple pattern geometries, which we have named centered, column, small and large cluster patterns. For the case of the column arrangement, the reviewer derived analytical expressions for $\langle d_{ry} \rangle$ and $\langle d_{ry} \rangle / \langle d_{yr} \rangle$ in terms of the class ratio, ϕ. The reviewer is concerned that their analytical expression for the mean shortest distance appears not to fit with the data we presented in Figure S3. In fact, this apparent discrepancy is because the data in Figure S3 represent normalised mean shortest distances. We normalise each mean shortest distance value by the same value computed in the null-model where colours are reassigned to nodes uniformly at random, preserving their relative abundance. For example, in our notation the normalised mean shortest distance from red to yellow nodes, $\widetilde{d}_{ry}$, is

$$\widetilde{d}_{ry} = \frac{\langle d_{ry} \rangle}{\langle d_{ry}^{null} \rangle}, \tag{1}$$

where $\langle d_{ry} \rangle$ and $\langle d_{ry}^{null} \rangle$ denote the mean shortest distance for the pattern and its null-model counterpart respectively. This is line with the approach used consistently throughout the paper for our mean first passage time calculations. We explain our method for computing the (normalised) mean shortest distance in the Methods section entitled "Mean shortest distance calculations" (lines 729-738 in the marked-up version of the revised manuscript), where we define the method and the notation used. We apologise if our previous formulation led to this misunderstanding and have edited the captions for Figures S3 & S4 to emphasise that we present normalised mean shortest distance values in these figures.

In this reply, we confirm that our results are consistent with the expressions derived by the reviewer, which describe the un-normalised mean shortest distance values in the column arrangement. By re-plotting the mean shortest distance data presented in Figure S3 of our manuscript without normalisation (Fig. A, top row), we show that these values agree with the expression derived by the reviewer. The slight deviations of our computed values from the analytical prediction for ϕ close to 0 and 1 are due to the discretisation of space. In the 54×54 discrete lattice, used for all of our column patterns, it is not always possible to exactly achieve the column pattern at such extreme values of ϕ, leading to slight deviations from the analytical result at these values.

We also plotted the values of the null model calculations, used for normalisation, to give further insight into the nature of the data plotted in Figure S3. These reveal how $\langle d_{ry}^{null} \rangle$ is reasonably constant for $\phi > 0.5$, and diverges for $\phi > 0.5$, which explains why $\widetilde{d}_{ry}$ for column patterns, in Figure S3, is maximal for some intermediate value of ϕ, and begins to decrease again as ϕ is increased past this value.

The reviewer also explained their intuitive expectation for the behaviour of $\langle d_{ry} \rangle$ and $\langle d_{ry} \rangle / \langle d_{yr} \rangle$ in the small clusters case. As with our analysis of the column patterns, we

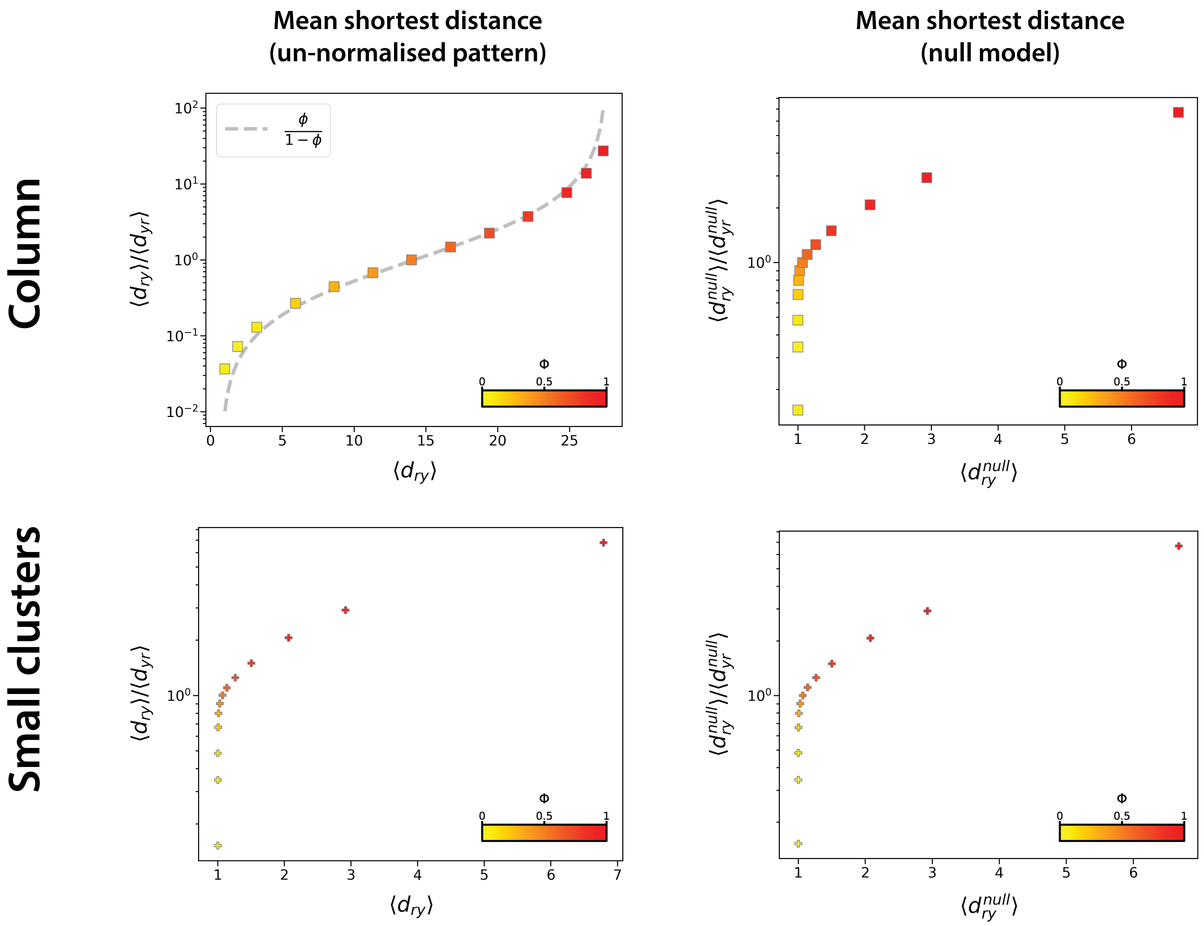

Fig. A: Mean shortest distance for column and small clusters patterns. Un-normalised mean shortest distance (left) and corresponding null model values (right) for column and small clusters arrangements. Points are coloured according to their class ratio, ϕ. Un-normalised mean shortest distance from red to yellow cells is denoted $\langle d_{ry}\rangle$, and from yellow to red denoted $\langle d_{yr}\rangle$, and the corresponding values computed in the null model are denoted $\langle d_{ry}^{null}\rangle$ and $\langle d_{yr}^{null}\rangle$ respectively.

re-plotted the mean shortest distance data for the small cluster patterns from Figure S3 without normalisation (Fig. A, bottom row). We again show that the values of $\langle d_{ry}\rangle$ and $\langle d_{ry}\rangle/\langle d_{yr}\rangle$ are in line with the reviewer's expectation. As the reviewer points out, $\langle d_{ry}\rangle/\langle d_{yr}\rangle < 1$ and $\langle d_{ry}\rangle \approx$ constant for small ϕ. Since the small cluster pattern has the same geometry as the null-model patterns, the resulting normalised mean shortest distance values for the small cluster patterns, $\widetilde{d}_{ry}$ and $\widetilde{d}_{ry}/\widetilde{d}_{yr}$, are constant and equal to 1 for all values of ϕ.

In summary, we have shown that the data presented in Figure S3 are correct and in agreement with the intuition of the reviewer, and the analytical results they have derived. We would like to reassure the reviewer that we employed the same high level of care and rigour throughout the entire manuscript as we did with these mean shortest distance calculations. We have clarified the captions of Figure S3 & S4 to emphasise that the data presented in these figures are normalised mean shortest distances.

Reviewer #2 comment

Column case

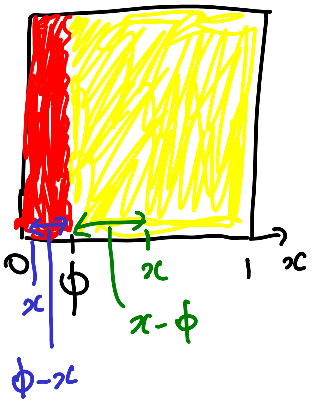

(here distances are in units of the total domain lenght. One fixed conversion factor to the results in the manuscript)

$$\tilde{d}_{ry} = \frac{1}{\phi}\int_0^{\phi} (\phi - x)\,dx = \frac{1}{\phi}\int_0^{\phi} u\,du = \frac{\phi^2}{2\phi} = \frac{\phi}{2}$$

$$\tilde{d}_{yr} = \frac{1}{1-\phi}\int_{\phi}^{1} (x-\phi)\,dx = \frac{1}{1-\phi}\int_0^{1-\phi} u\,du = \frac{(1-\phi)^2}{2(1-\phi)} = \frac{1-\phi}{2}$$

prediction for figure S3, right panel:

$$x = \tilde{d}_{ry} = \frac{\phi}{2}$$

$$y = \frac{\tilde{d}_{ry}}{\tilde{d}_{yr}} = \frac{\phi}{1-\phi} = \frac{2x}{1-2x}$$

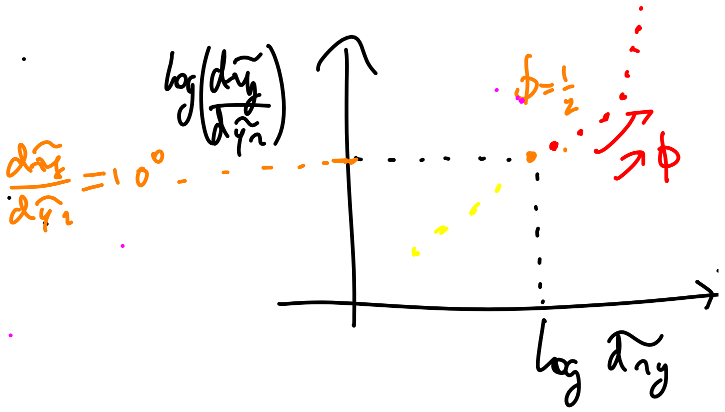

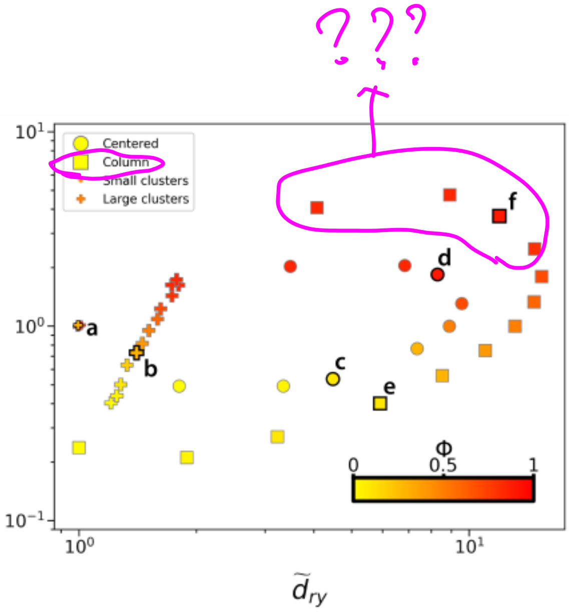

Small cluster case:

(assuming small red cluster in yellow domain, with density of clusters ↗ with φ)

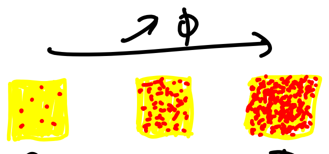

$\tilde{d}_{ry}$: mainly controlled by cluster size, thus ≈ constant.

$\tilde{d}_{yr}$: $> \tilde{d}_{ry}$ … $\simeq \tilde{d}_{ry}$

$\frac{\tilde{d}_{ry}}{\tilde{d}_{yr}} < 1$ … $\frac{\tilde{d}_{ry}}{\tilde{d}_{yr}} \simeq 1$

$\frac{d\tilde{r}_y}{d\tilde{r}_x}$ should be <1 for ϕ small

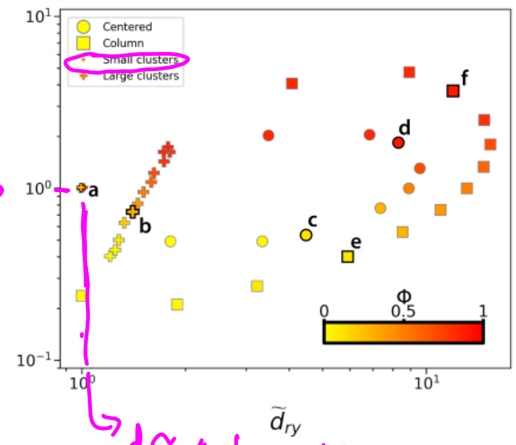

$d\tilde{y} \approx ct$: OK.
