# Supplementary material for: First passage time analysis of spatial mutation patterns reveals sub-clonal evolutionary dynamics in colorectal cancer
Source: PLoS Comput Biol. 2023 Mar 13;19(3):e1010952. doi: 10.1371/journal.pcbi.1010952 (PMC10035892; doi:10.1371/journal.pcbi.1010952)
Supplement: Attachment — Submitted filename: reviewerComments_responses.pdf [file pcbi.1010952.s038.pdf]

All line references in this response letter are made with respect to the marked-up copy of the revised manuscript (revised_manuscript_marked_up.pdf)

Reviewer #2: *First, I wish to thank the authors for the explanation. I had missed that for the shortest distance, it is also renormalized, and I find it very reassuring to see that then the simulation results are as expected from analytics in the simple cases for which it is possible to calculate what is expected.*

Going back to the rest of the 1st re-submission, I thank the authors for their extensive revision work. There are some interesting additions to the manuscript. I would say that I am still not fully convinced of the usefulness of the method proposed compared to alternatives, but given that the claims are toned down compared to the first draft, and that some points are now clearer, I think that this exploration of this new method is worth publishing. I have a few more comments, but small revisions are enough.

Here, the detailed comments in 3 parts. First, the elements which makes me unconvinced, and thus justifying to tone down a bit more the text. Second, some specific elements that still feel like overselling. Third, other small comments, more or less in the order of the text.

We thank the reviewer and the editors for their careful consideration of our manuscript and the time taken to review and comment on our work. In light of the reviewer's latest comments, we have toned down various elements of the manuscript, and have responded point-by-point below. We hope that the revised manuscript is to the satisfaction of the reviewer and the editors.

1/ First, about why I am not totally convinced by the answers to my concerns:

In the response to the reviews, in regard to the CMFPT vs. shortest distance, the authors state that "We agree with the reviewer and show that the results using mean shortest distance are similar to those derived using the CMFPT (Supplemental figures 3 and 4 in the revised manuscript). We argue, however, that the CMFPT conveys slightly more information about the pattern in question, due to the process of finding the distribution of distances from cell type A to B using a stochastic process, compared to the deterministic process of finding the shortest distance (i.e. the approach of the mean shortest distance metric). This means that the CMFPT enables us to obtain a distribution of transition times for every starting node in the network, unlike the mean shortest distance, and therefore conveys more information about the neighborhood of the starting cell." Indeed to calculate the CMFPT, there are many trajectories calculated, and thus a distribution. But then in the comparison with simulations of the process, only the mean is computed. Thus no more information is conveyed, though the information conveyed is slightly different. One would need another measure to compare with the simulations, for instance the variance of the first passage time, to really use the distribution. And indeed in the simple examples, there is very little difference in the patterns between CMFPT and shortest distance. Given that using the CMFPT instead of the shortest distance does not seem to affect much how well patterns can be distinguished (see figure 2 vs figure S3; and figure 4 vs. figure S4), using a technique that is more computationally intensive and potentially noisy, is not clearly justified.

The reviewer raises a legitimate issue regarding our comparison of CMFPT to the mean shortest distance. In its current form, we only summarise the first passage time distributions using the mean, and thus should not claim that our method offers more information about the geometry of the sub-clonal patterns than mean shortest distance. Accordingly, we have toned down the text between lines 142-145 where we compare CMFPT to the mean shortest distance. Here, we also describe how future implementations of our method, utilising higher order moments of the first passage time distributions, could offer a different description of the pattern geometry than the mean shortest distance by capturing information about the neighbourhood of each cell. Furthermore, we have added new discussion between lines 623-633 where we offer further justification for the development and use of first passage time methods, highlighting their future potential to quantify the cell neighbourhoods by exploiting the distribution of first passage times.

The answer about scales is not really satisfying, because actually (sometimes small) subparts of the tumors are compared, not the whole image of the 8000micronsx8000microns field. Thus comparing bits of different sizes to always simulations with the same N total does not seem right. It is actually less important than the 3D vs. 2D issue (which also make for different Ntot).

One strength of the CMFPT method is that, given that we work with normalised values, it is possible to compare patterns which differ in size. Whilst we endeavoured to compare simulated and experimental patterns containing a similar number of cells, our method of comparing data is perhaps not optimal, and it is possible that we lose information by not matching the size of the compared patterns. A modified simulation model, for which parameter N_{max} is allowed to vary, would be an interesting direction for future research, and could be a more adaptable method of comparing experimental and simulated patterns. Alternatively, keeping parameter N_{max} as constant, but extracting subregions of our simulated patterns (the size of which could be matched to the size of the experimental image in question) could also be explored a future study. We thank the reviewer for prompting us to think in this direction and have toned down the text between lines 246-254 to acknowledge these points and make clear to our readers that this is an area of potential improvement in our method.

I also do not understand the (arbitrary?) choice of subparts of the tumors to be compared. Indeed, the results are showing different inferred parameters within the same slice.

To select the subparts of the experimental images, we manually chose regions which contained connected wild-type and sub-clonal tumour populations. The motivation for this was to reduce the influence of non-tumour regions between the tumour populations on our CMFPT measurements and is outlined between lines 269-274. Since our experimental tissue samples are, themselves, subparts extracted from larger 3-dimensional tumours, we believe it is justified to extract smaller subparts from the samples in order to explore how to optimise our analysis. Furthermore, given the complexity of the biological systems we are analysing, and the stochastic nature of our model and analysis method, it is not wholly unsurprising that we obtain different inferred parameters within the same slice, in samples where we extracted multiple subparts. We offer some explanation for the fluctuating values of the inferred parameters between lines 367-380 where, alongside errors associated to our analysis, we suggest that spatial variations in local tissue density, access to nutrients, and proximity to the immune system could also affect the perceived dynamics within individually analysed subparts.

The simulations with similar rescaled CMFPT give sometimes very different patterns. See for instance figure 6: [1] : phi is completely off. [4] and patterns on the left: the yellow area much more fragmented in real samples than in the simulation: could be strong indication of multiple mutations for instance, or transport not consistent with model used here.

The best-fit model pattern assigned to [1] in figure 6 is an example of an experimental sub-clonal pattern falling into a region of the CMFPT phase space shared by model patterns from several different parameter combinations. Specifically, when considering the set of all simulated tumour CMFPT values in figure 4, one can observe that the data fall onto one of two “arms” *i.e.*, a lower and upper “arm” in the vertical axis. The upper arm consists of tumours with either low n_{mut} or high s (or both), whilst the lower arm tends to contain tumours with higher n_{mut} or low s. Occasionally, as is the case with sub-region [1] from figure 6, experimental images fall into the region between the lower and upper arm and can be erroneously interpreted as consistent with model patterns from (probably) the wrong arm. Since the patterns lying on the upper and lower arms have vastly different model parameters, those experimental patterns which are erroneously classified will be ascribed a best-fit model image with a vastly different phi. The variance of the CMFPT data in the upper arm tends to be rather low for each parameter combination, and so these errors do not happen very often, however occasionally this scenario can happen. We have addressed and explained this between lines 318-326.

We agree that the second case referenced by the reviewer, of sub-region [4] in figure 6, could be an indication of multiple mutations. It is true that parallel evolution has been observed in clear-cell renal cell carcinoma, and in non-small cell lung cancer. Yet, evidence of parallel evolution in colorectal cancer is limited. In this experimental data, mutated regions were tested for using mutation-specific RNA probes, and no samples were analysed with multiple probes. This means that cells in the fragmented mutated regions carry the same point mutation in the same genomic position. Since is possible but extremely unlikely to have cells mutating independently in precisely the same genomic location, we assumed that all observed mutants in any one sample must have originated from the same mutational event. If multiple independent events were to have occurred in the samples we analysed, however, it could explain some of the observed non-contiguous regions of mutant cells. Our computational model does not account for multiple occurrences of the same mutation but could easily be adapted to do so.

Another explanation for the observed spatially disparate regions of mutant cells is that they are in fact part of a single, contiguous mutant population in the 3D tumour, yet appear spatially separated due to the orientation of the 2D sample. Since there is limited evidence for parallel evolution in colorectal cancer, yet we know for certain that our experimental samples were acquired from 3-dimensional tumours, we assume that the mutant clade arose from a single founding mutant cell, and that the observed patterns can be explained by varying strengths of cell pushing coupled to 3D sampling effects. As the reviewer also points out, these fragmented mutant regions could also possibly be explained by modes of cell transport other than the pushing mechanic we implemented in our model. For example, short range cell migration could potentially produce these observed patterns. We have added some discussion on this topic, as well as the effects of multiple mutations, between lines 487-509.

Finally, the manuscript makes clear a series of limitations (2D vs 3D, not using for now the additional information of phi, limit in the number of simulated parameter values (e.g. q), dynamics of the non-cancerous cells omitted) which will make hard to apply such a method, but these explanations of the limitations are actually a good point of the present manuscript.

2/ Here a list of places where the claims should be toned down:

- in the abstract "We uncover a wide range of sub-clonal dynamics" > "We infer a wide range of sub-clonal dynamics" (there is no guarantee that the results are actually what happened in the tumor)

This is a fair point and we have implemented the suggested change in language in the abstract.

- around line 148: "Mean shortest distance is a deterministic measure of the distance between cells, whereas CMFPT constructs

paths between pairs of cells using a stochastic process, resulting in a distribution of first passage times for each starting cell. As such, CMFPT provides more information about the texture of the patterns than mean shortest distance by incorporating information about the neighbourhood of each cell, and gives a better description in more complex patterns and experimental data." Given only the mean is used, and that the supplementary figure with the shortest distance is very similar to the one wit the CMFPT, there is no clear reason that "more" information is given. The last sentence could be changed to something like "As such, CMFPT may provide a different information about the texture of the patterns than the mean shortest distance, by incorporating information about the neighborhood of each cell. "

We agree with the reviewer that, given the data we present, we cannot substantiate our claims that the CMFPT provides more information than the mean shortest distance. We focussed on using only the mean to summarise the CMFPT distributions but, in principle, one can use higher order moments of these distributions, such as variance, to obtain further information about each cell's neighbourhood. We have edited the text between lines 142-145 to acknowledge these points.

- around line 226: "it is possible to recover details about the underlying cell by quantifying the resulting spatial patterns alone." : a bit too optimistic, as there are several sets of parameters giving similar results: "it is possible to narrow down the potential underlying cell dynamics by quantifying the resulting spatial patterns alone."

We thank the reviewer for this comment and have changed the text as suggested at line 219.

- After figure 6 there should be at least a sentence commenting that some patterns of the simulations are actually not consistent with the actual sample, despite having similar CMFPT.

This can occur due to the size of the variance in the distribution of CMFPT values for certain model parameters, and there being an overlap between these distributions for different combinations of parameters. We have acknowledged and explained this between lines 318-326.

3/ Other comments

- how many trajectories to compute the CMFPT?

We run 5,000 trajectories for each starting node, average over them to get the mean first passage time (MFPT) for each node and then average over all the nodes of the same class to obtain the class mean first passage time (CMFPT). We have added this technical note to the Methods section titled "Simulating random walkers and estimating first passage time statistics" (lines 664-668).

- around line 75 : why mention tau yy and tau rr? Never used afterwards, and their definition will be more sensitive to the chosen subsampled spatial grid.

To visualise the CMFPT data throughout the manuscript, we plot the data in the phase space spanned by $\tilde{\tau}_{ry}$ and $(\tilde{\tau}_{ry}/\tilde{\tau}_{yr})$. Our motivation for this choice of phase space is explained in the manuscript between lines 82-85. When performing parameter inference, however, we compare the distance between pairs of patterns in the 4-dimensional phase space spanned by $\tilde{\tau}_{yy}$, $\tilde{\tau}_{yr}$, $\tilde{\tau}_{ry}$ and $\tilde{\tau}_{rr}$. We outline this approach in the Methods section titled "Bayesian grid-search for best-fit model parameter inference" (lines 737-740) but have now further clarified this detail in the caption of figure 5 in the attached revised manuscript. Whilst $\tilde{\tau}_{rr}$ and $\tilde{\tau}_{yy}$ may be more sensitive than $\tilde{\tau}_{ry}$ *and* $\tilde{\tau}_{yr}$ to the choice of subsampled spatial grid we find that, since we simulate many trajectories for each node in the image and compute the mean over all nodes, that these quantities are reliable and do offer some small extra insight into the geometry of the patterns alongside $\tilde{\tau}_{yr}$ and $\tilde{\tau}_{ry}$.

- legend of figure 4: no description of the smaller figures

We thank the reviewer for drawing attention to this omission and have added a description of the inset plots to the legend of Fig 4.

- figure 4 : I suppose the smaller figures show a point for each simulation, with 100 simulations for each symbol of the larger figure, (with color for the final phi). There seem to be quite some spread in some cases. It would be interesting to show the typical range of outcomes for a given q,s,nmut, for instance with errorbars on the symbols. That may be too much for this figure that is already busy, but one may want to keep this in mind when comparing simulations and experimental images.

We agree that there is a reasonable spread in the data for certain parameter combinations. Whilst it may be too busy to include in Fig 4, we have plotted this data separately in a new figure in the supporting information (S9 Fig.). We have also added some text between lines 293-296 to acknowledge the spread in the data, and overlapping distributions, for certain model parameters.
